# cPRIME enables endogenous mapping of genetic variant effects at RNA level

**DOI:** 10.1038/s41467-026-78344-9

**Published:** 2026-10-15

**Authors:** Ghanem El Kassem, Constanze Westphal, Marvin Doebel, Jasmine Hillmer, Tanja Seifert, Michael Boettcher

**Affiliations:** Institute of Molecular Medicine, Section for Molecular Medicine of Signal Transduction, Faculty of Medicine, Martin Luther University Halle-Wittenberg, Halle, Germany

**Keywords:** Mutagenesis, CRISPR-Cas9 genome editing, Cancer genetics, Gene expression profiling

## Abstract

Understanding the functional consequences of genetic variants, particularly those classified as variants of uncertain significance (VUS), is a major challenge in genomics. While variant effects at protein level are well recognized, roles in post-transcriptional regulation are increasingly appreciated. Here, we introduce cPRIME (prime editing with cDNA readout), a scalable method for assessing variant effects at RNA level. cPRIME employs prime editing to insert genetic variants into endogenous genomic loci, followed by the quantification of changes in variant frequencies at RNA (cDNA) and genomic DNA (gDNA) level. We demonstrate its effectiveness across diverse karyotypes and genomic loci and reveal that synonymous marker edits - frequently used in prime editing screens - can themselves alter RNA processing and thereby confound the interpretation of variant effects. Taken together, cPRIME provides a platform for studying the post-transcriptional consequences of genetic variants in their genomic context and establishes a framework for the functional interpretation of RNA-altering variants in cancer and other diseases.

## Abstract (web summary)

Understanding how genetic variants affect RNA is critical for interpreting disease-associated variants. Here, the authors introduce cPRIME, a scalable prime editing-based approach to introduce variants into the genome and systematically assess their effects at the RNA level.

## Introduction

The advent of next-generation sequencing (NGS) technologies in the mid-2000s has revolutionized genomics by enabling the high-throughput identification of genetic variants^1^. While the effects of nonsense variants that introduce premature stop codons are generally considered disruptive to the function of the encoded protein, the functional consequences of many other identified variants—collectively referred to as variants of uncertain significance (VUS)—remain unknown^2,3^. Over time, it has become increasingly evident that even variants which do not alter the amino acid sequence of the encoded protein—so-called synonymous variants—and variants in non-coding regions of protein-coding genes, such as introns or untranslated regions (UTRs), can exert functional effects by impacting RNA processing and stability rather than protein function^4,5^. Therefore, a deeper understanding of how variants in both coding and non-coding regions influence post-transcriptional regulation is critical to uncovering their roles in gene regulation and disease mechanisms.

In the past, two key methods—Massively Parallel Reporter Assays (MPRA) and Saturation Genome Editing (SGE)—were used to study the effects of genetic variants on RNA level at scale. MPRA allows for the systematic testing of variants outside of their genomic context by introducing variant libraries into reporter constructs^6–9^. While MPRA provides valuable insights, it is limited in that it does not evaluate genetic variants within their endogenous context, making it less relevant for studying variant effects on native gene regulation. SGE, on the other hand, is a method to assess variant effects within their genomic context. It relies on a homology-directed repair (HDR)-based approach using CRISPR/Cas9 to introduce precise variants into the genome of cultured cells, and has been used to study variant effects, including effects on RNA level^10,11^. However, SGE suffers from limitations such as low HDR efficiency in many cell types and the need for separate donor libraries for each 200 bp genomic region^12^.

To address the limitations of MPRA and SGE, we introduce cPRIME—prime editing with cDNA readout—a method for assessing genetic variant effects on RNA. Unlike previous techniques, cPRIME leverages the precision of prime editing to introduce variants into their endogenous genomic locus without causing double-strand breaks or requiring donor DNA templates^13^. Prime editing enables all possible base conversions, as well as short insertions and deletions, making it ideal for the investigation of variant effects. cPRIME follows a pooled screening strategy, where genetic variants are introduced into the genome of host cells, and the effects at the RNA level are assessed by quantifying editing frequencies at both the genomic DNA (gDNA) and complementary DNA (cDNA) levels. The ratio of cDNA to gDNA variant frequencies provides a direct measure of post-transcriptional effects at the RNA level, enabling identification of variants that affect RNA splicing and abundance.

Importantly, we demonstrate that cPRIME functions across multiple genomic loci in both near-haploid (KBM7) and near-triploid (K562) cell lines, highlighting its applicability across diverse karyotypes. We further show that synonymous marker edits—frequently used in prime editing screens to improve sensitivity^14–17^—can themselves alter RNA processing. These findings highlight the need for careful design and computational evaluation of future pegRNA libraries incorporating synonymous marker edits.

In conclusion, cPRIME offers a scalable method for studying the effects of endogenously introduced genetic variants at the RNA level. We anticipate that cPRIME will become a valuable tool for dissecting post-transcriptional variant effects in both coding and non-coding RNAs. By linking genetic variation to RNA level outcomes, cPRIME can help deepen our understanding of disease mechanisms in cancer and other genetic disorders.

## Results

### Synonymous marker edits enable the detection of rare prime editing events

Throughout this study, we used two chronic myeloid leukemia cell lines: near-haploid KBM7 and near-triploid K562. We performed pooled prime-editing screens targeting *NF1*, a Ras-GAP frequently mutated in cancer. We first designed and cloned a pegRNA library to introduce variants reported in cBioPortal^2^ into exon 27 of *NF1*, which contributes to the central GTPase-activating protein-related domain (GRD). This included 38 missense variants and 12 nonsense variants (stop codons). Additionally, for each codon, we analyzed whether a single-nucleotide substitution at any of its three positions could generate a stop codon and designed pegRNAs accordingly, adding 9 pegRNAs to the library. Synonymous edits were also included as controls for each codon variant, contributing 39 pegRNAs. Altogether, the library consisted of 98 pegRNAs.

**Fig. 1:**
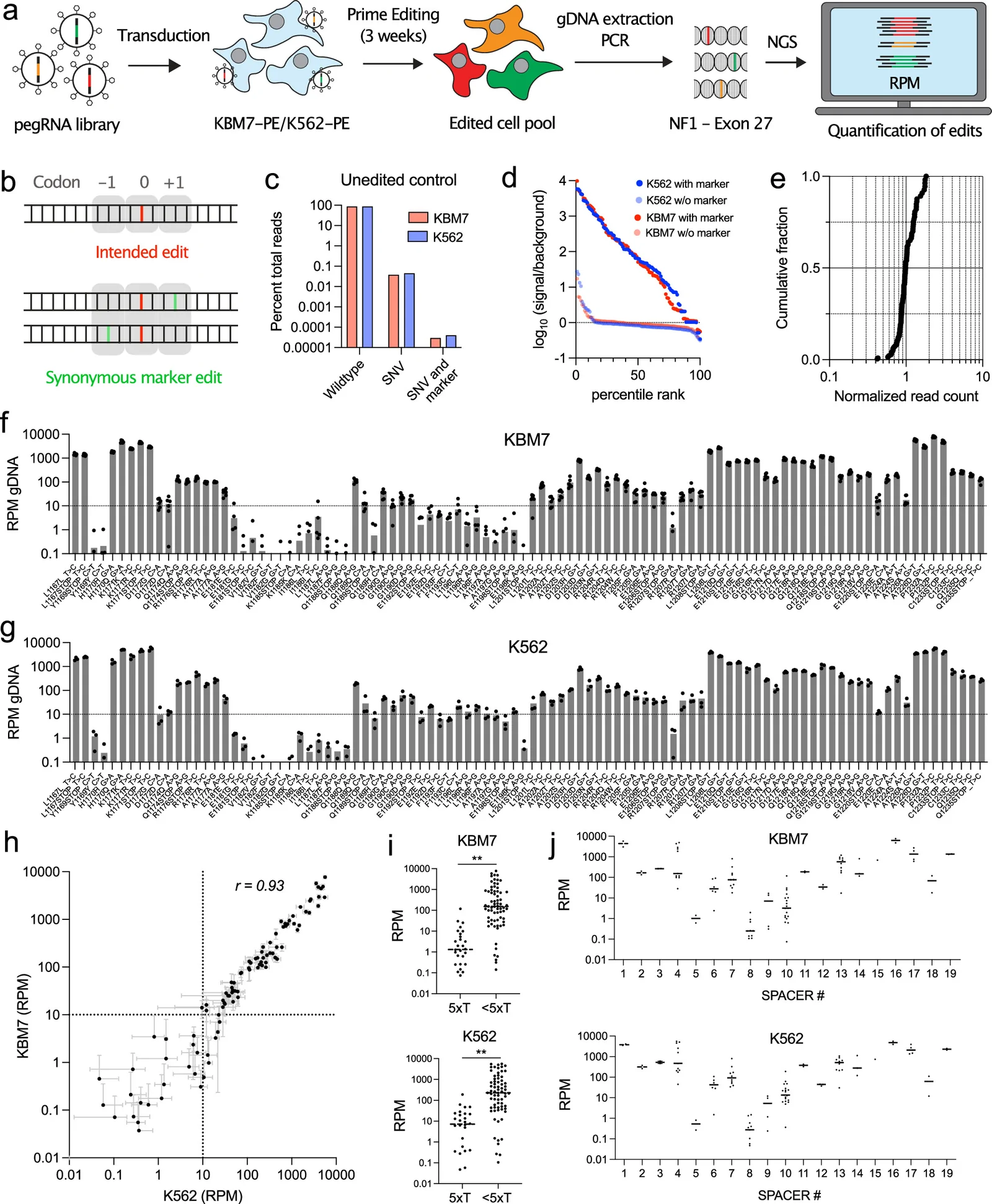
Synonymous marker edits enable the accurate quantification of low-frequency prime editing events. **a** Overview of pooled prime editing screens to introduce genetic variants into exon 27 of *NF1*. RPM: reads per million. **b** Synonymous marker strategy. **c** Frequency of SNVs without and with marker edit in unedited control samples (background). **d** Signal-to-background ratios from prime editor screens with and without (w/o) synonymous marker edits. Odds ratio was calculated using Fisher’s exact test. **e** Distribution of the *NF1* exon 27 pegRNA library used throughout this study. The x-axis shows the normalized read count of each pegRNA (raw count divided by the mean count across all pegRNAs). The y-axis shows the cumulative fraction of pegRNAs (each count divided by the summed counts of all pegRNAs, accumulated from lowest to highest). **f** Prime editing frequencies as reads per million (RPM) in KBM7 cells at 21 days post-transduction of the pegRNA library and **g** K562 cells at 45 days post-transduction. Combined edit-marker variants are denoted as the intended amino acid change followed by the nucleotide substitution introduced as a synonymous marker. Values represent the mean of biological replicates; *n* = 3. **h** Correlation of editing frequencies between KBM7 and K562 cells. Values represent the mean of biological replicates; error bars, SD (*n* = 3). Pearson correlation was used to determine the *r* value. **i** Editing frequencies from pegRNAs with five consecutive thymine bases (5xT) and pegRNAs with less than five consecutive thymine bases (<5xT). Unpaired two-tailed t-test; KBM7 *P* value = 0.006; K562 *P* value = 0.002. Values represent the mean of biological replicates (*n* = 3). **j** Editing frequencies separated by different pegRNA spacer sequences. Values represent the mean of biological replicates (*n* = 3). Source data are provided as a Source Data file.

The screen workflow is illustrated in Fig. 1a. Lentiviral transduction of the pegRNA library was carried out in KBM7 and K562 cells stably expressing the prime editor (nCas9-RT, PE2, as described by Anzalone et al.^13^). Deletion of MLH1 in both cell lines, a modification shown by Chen et al. to enhance prime editing efficiency^18^, upgraded the system to PE4. After editing, genomic DNA was extracted, *NF1* exon 27 was amplified by PCR, and amplicons were sequenced to quantify editing frequencies as reads per million (RPM).

In pooled pegRNA screens, low-frequency editing events can be difficult to distinguish from PCR amplification or sequencing errors, particularly for SNVs, which differ from the wild-type sequence by only one nucleotide. To improve discrimination of true edits from background, we incorporated synonymous marker edits adjacent to the intended SNV edits (Fig. 1b), a strategy independently developed by multiple groups^14–17^. In unedited control cells, edit-level background frequencies were 0.037 ± 0.041% in KBM7 cells and 0.045 ± 0.049% in K562 cells, consistent with the expected contribution of PCR amplification and sequencing errors (Fig. 1c). By contrast, the background frequency of the combined edit-marker haplotype in unedited controls was 1280-fold lower in KBM7 cells and 1120-fold lower in K562 cells. This reduced background allowed marker-linked edits to be quantified above the background threshold for nearly 90% of variants, compared with approximately 10% of SNVs quantified without marker edits (Fig. 1c, d and Supp. Data 1–3).

### Prime editing frequencies are highly variable across target sites

Next, we used the *NF1* exon 27 library to conduct prime editing screens as described in Fig. 1a. While the distribution of the pegRNAs in the library was very narrow (Fig. 1e and Supp. Data 4), the detected editing frequencies varied over several orders of magnitude in both cell lines, KBM7 (Fig. 1f, and Supp. Data 1) as well as K562 (Fig. 1g and Supp. Data 2). Of the 98 intended edits, 69% (KBM7) and 76% (K562) were detectable at a threshold above 10 RPM. Without the incorporation of synonymous marker edits, these edits would have been indistinguishable from the background, as the observed error rates (Fig. 1c and Supp. Data 1, 2) would translate to approximately 370 RPM from unedited KBM7 cells and 450 RPM from unedited K562 cells. Editing efficiencies were highly target site specific and consistent across cell lines (Fig. 1h). One explanation for the differences in editing efficiencies seems to be the presence of >4 consecutive thymine residues in the pegRNA sequence (Fig. 1i), an observation that was also recently made by Oh et al.^16^. Since guide RNAs, including pegRNAs, are typically expressed from polymerase III promoters (here human U6), this finding is not surprising, as poly-T is a known polymerase III stop signal. Another determinant for prime editing efficiency seems to be the 20 nt spacer sequence of the pegRNA. The analysis of editing frequencies from all 98 pegRNAs contained in the library, separated by spacer sequences (Fig. 1j), revealed that pegRNAs with certain spacers (e.g. #3 and #8) resulted in very low editing frequencies, while others reproducibly allowed high editing efficiencies (e.g. #16, #17 and #19).

In summary, our findings demonstrate that synonymous marker edits enable precise quantification of rare editing events. Moreover, poly-T stretches and spacer sequences are critical determinants of prime editing efficiency, offering insights for future optimization.

### cPRIME enables the systematic mapping of variant effects at RNA level

**Fig. 2:**
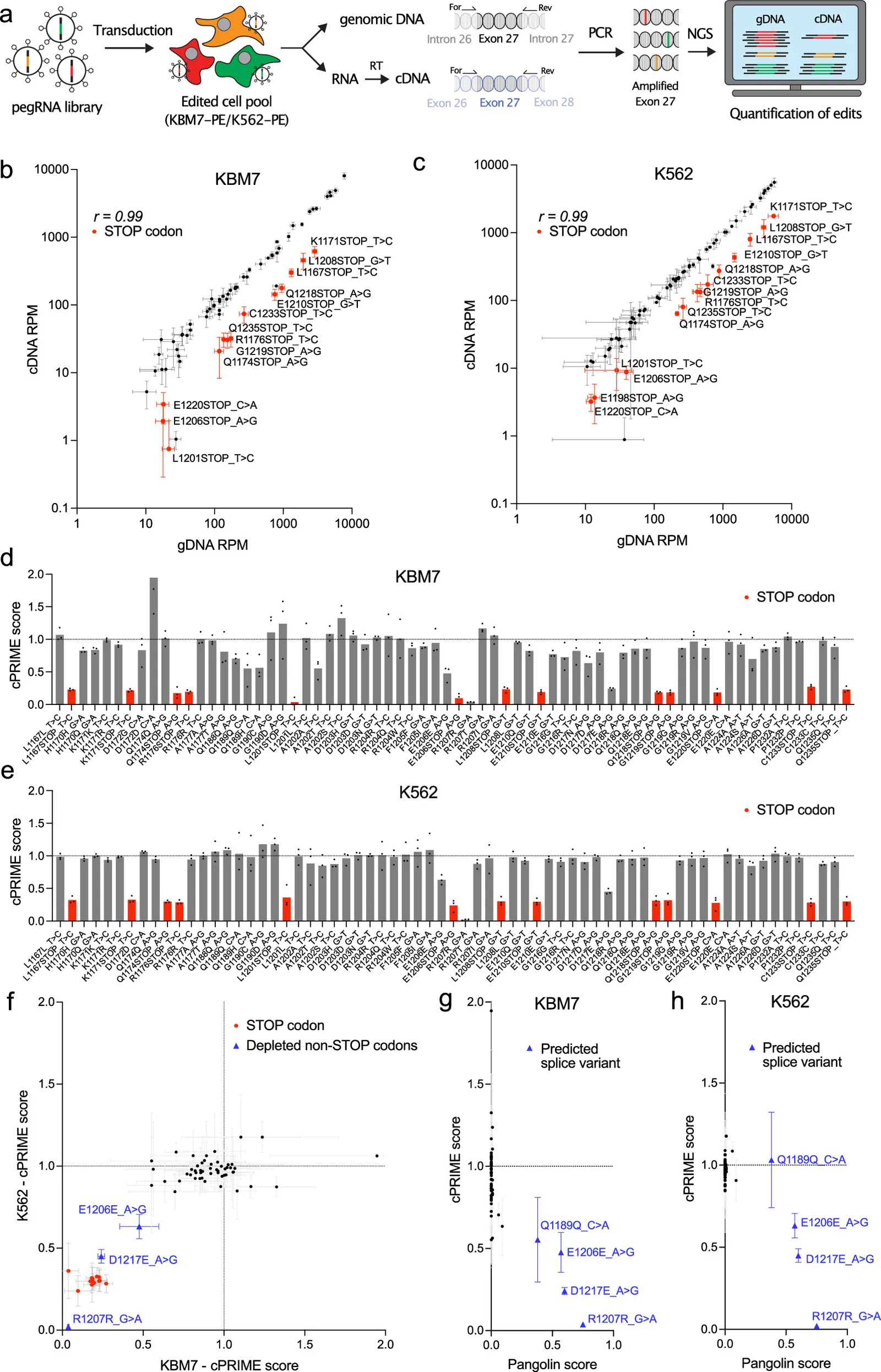
cPRIME enables the systematic mapping of genetic variant effects at the RNA level. **a** An overview of the cPRIME strategy. Exon 27 is amplified via PCR from gDNA (primer binding sites in intron 26 and intron 27) and cDNA (primer binding sites in exon 26 and exon 28). RT: reverse transcription. **b** Editing frequencies as reads per million (RPM) at genomic DNA (gDNA) versus RNA (cDNA) level in KBM7 and **c** K562 cells. Nonsense (STOP) variants are shown in red. Pearson correlation was determined between non-STOP variants only. Values represent the mean of biological replicates; error bars, SD (*n* = 3). **d** cPRIME scores in KBM7 and **e** K562 cells. Nonsense variants are shown in red. Values represent the mean of biological replicates; *n* = 3. **f** Correlation of cPRIME scores between KBM7 and K562 cells. Nonsense variants are shown in red. Predicted splice variants are shown in blue. Values represent the mean of biological replicates; error bars, SD (*n* = 3). **g** Correlation between Pangolin scores and cPRIME scores in KBM7 and **h** K562 cells. Nonsense variants are shown in red. Predicted splice variants are shown in blue. Values represent the mean of biological replicates; error bars, SD (*n* = 3). Source data are provided as a Source Data file.

To investigate the effects of genetic variants at the RNA level, we developed an approach which we termed cPRIME - prime editing with cDNA readout. As shown in Fig. 2a, cPRIME involves simultaneous measurement of editing frequencies at both the genomic DNA (gDNA) and RNA (cDNA) levels. As above, editing frequencies were quantified as RPM. Overall, we observed a strong correlation between gDNA and cDNA editing frequencies in both KBM7 (Fig. 2b and Supp. Data 1) and K562 cells (Fig. 2c and Supp. Data 2). All edits introducing nonsense (STOP) variants showed lower RPM at the cDNA level compared to the gDNA level, consistent with mRNA degradation via nonsense-mediated decay^19^. As a result, the cPRIME scores - calculated as the cDNA/gDNA RPM ratios - for all nonsense (STOP) variants were <1, with averages of 0.19 ± 0.06 for KBM7 (Fig. 2d) and 0.30 ± 0.03 for K562 (Fig. 2e). On the other hand, most non‑STOP variants showed cPRIME scores close to 1, with the exception of variants E1206E_A > G (edit: c.3618 G > A, marker: c.3621 A > G), R1207R_G > A (edit: c.3621 A > G, marker: c.3624 G > A) and D1217E_A > G (edit: c.3651 T > G, marker: c.3654 A > G), which consistently showed cPRIME scores <1 in both cell lines (Fig. 2f).

It is important to point out that reduced cPRIME scores can originate from distinct post-transcriptional mechanisms. Because the cDNA amplicon is generated with primers in the neighboring exons and is size-selected by gel purification before sequencing, a variant-bearing transcript is counted only if it preserves its search sequence (the edited sequence and adjacent 10 bp up and downstream of the wild-type sequence), both flanking primer binding sites, and an approximately canonical amplicon size. While nonsense variants decrease transcript abundance most likely through nonsense-mediated decay, splice-altering variants may generate aberrantly processed transcripts that fail one or more of these criteria and are thus underrepresented in the cDNA readout. As a result, variants affecting canonical RNA splicing or transcript abundance can show reduced cDNA/gDNA RPM ratios. Importantly, a reduced cPRIME score reflects depletion of the specifically detected isoform rather than necessarily a decrease in total transcript levels, and does not by itself reveal whether an alternative isoform is produced or whether such an isoform is functional.

To further investigate the reduced cPRIME scores observed for non-STOP variants, we analyzed all non-STOP variants together with their corresponding synonymous marker edits using the splice prediction tool Pangolin^20^. Briefly, Pangolin is a deep learning-based model that predicts the impact of sequence variants on RNA splicing. It reports separate scores for predicted splice site gain (0–1) and loss (0–1), with values closer to ±1 indicating stronger effects. As the assayed variants were designed in regions where no splice site is expected, we focused on the splice gain score rather than the splice loss score. Pangolin assigned a splice site strength increase above 0.5 to all three non-STOP variants, showing decreased cPRIME scores when combined with their respective marker edit, suggesting the activation of cryptic splice sites (Fig. 2g, h and Supp. Data 5).

### Synonymous marker edits can influence RNA processing

To technically validate the pooled screen results, the variants E1206STOP_A > G (edit: c.3616 G > T, marker: c.3621 A > G), E1206E_A > G (edit: c.3618 G > A, marker: c.3621 A > G) and R1207R_G > A (edit: c.3621 A > G, marker: c.3624 G > A), and two control variants lacking screen phenotypes: R1207I_G > A (edit: c.3620 G > T, marker: c.3624 G > A) and R1207T_G > A (edit: c.3620 G > C, marker: c.3624 G > A) were introduced into KBM7 and K562 cells in an arrayed format. Each variant was combined with its corresponding synonymous marker edit used in the pooled screen. A high correlation between the screen and validation cPRIME scores confirmed that these edits impact RNA processing, as observed in the pooled screens (Supp. Fig. 1a, b and Supp. Data 6).

To further investigate the impact of synonymous marker edits on screen phenotype, we introduced three nonsense variants: R1176STOP_T > C (edit: c.3526 A > T, marker: c.3531 T > C), E1206STOP_A > G (edit: c.3616 G > T, marker: c.3621 A > G), and Q1218STOP_A > G (edit: c.3652 C > T, marker: c.3657 A > G), three negative-control variants lacking a screen phenotype: K1171K_T > C (edit: c.3513 G > A, marker: c.3516 T > C), R1204Q_T > C (edit: c.3611 G > A, marker: c.3615 T > C), P1232A_T > C (edit: c.3694 C > G, marker: c.3699 T > C), and three non-STOP variants leading to the strongest reduction in cPRIME scores: E1206E_A > G (edit: c.3618 G > A, marker: c.3621 A > G), R1207R_G > A (edit: c.3621 A > G, marker: c.3624 G > A), D1217E_A > G (edit: c.3651 T > G, marker: c.3654 A > G), each without their respective synonymous marker edits. While the nonsense and negative-control variants showed the expected phenotypes, only one of the three non-STOP variants - D1217E_A > G (edit: c.3651 T > G, marker: c.3654 A > G) - caused a reduction in cDNA reads, although to a lesser degree than observed with the corresponding synonymous marker edit in the screens (Supp. Fig. 1c, d and Supp. Data 6). Comparison of these findings to Pangolin predictions revealed that the splice gain score is completely lost upon removal of the marker variant for E1206E_A > G (edit: c.3618 G > A, marker: c.3621 A > G) (Pangolin splice gain score of 0.57 down to 0) and partly lost for R1207R_G > A (edit: c.3621 A > G, marker: c.3624 G > A) (0.75 down to 0.22), only D1217E_A > G (edit: c.3651 T > G, marker: c.3654 A > G) retained a large part of its predicted splice activity even after removal of the marker (0.6 down to 0.46) which closely mirrors our observations (Supp. Fig. 1c, d).

Finally, variant R1207R_G > A (edit: c.3621 A > G, marker: c.3624 G > A) was further investigated, as the most pronounced phenotype was observed in the pooled screens when it was combined with its synonymous marker edit (c.3624 G > A). The introduction of the variant alone did not cause a reduced cPRIME score in either of the two cell lines (Supp. Fig. 1e, f and Supp. Data 6). Neither was a reduced cPRIME score observed, when a cytosine or thymine (c.3624 G > C, c.3624 G > T) instead of the original adenine substitution was used to replace the endogenous guanine (c.3624 G > A) at the marker position in codon 1208 (Supp. Fig. 1e, f). Pangolin predictions largely followed our observations, where only the combination of our intended edit with its synonymous marker leads to a strong predicted splice site gain score of 0.75 while all other installed edits result in a splice gain score of ~0 to 0.2 (Supp. Fig. 1e, f). These observations indicate that certain, precise variant-marker combinations can dramatically influence RNA processing by introducing novel splice motifs - an effect that future pegRNA library designs should take into account. Splice-site predictors such as Pangolin may prove to be valuable tools for eliminating splice site-generating marker variants during pegRNA library design (Fig. 2g, h).

### cPRIME enables simultaneous analysis of multiple genomic loci

**Fig. 3:**
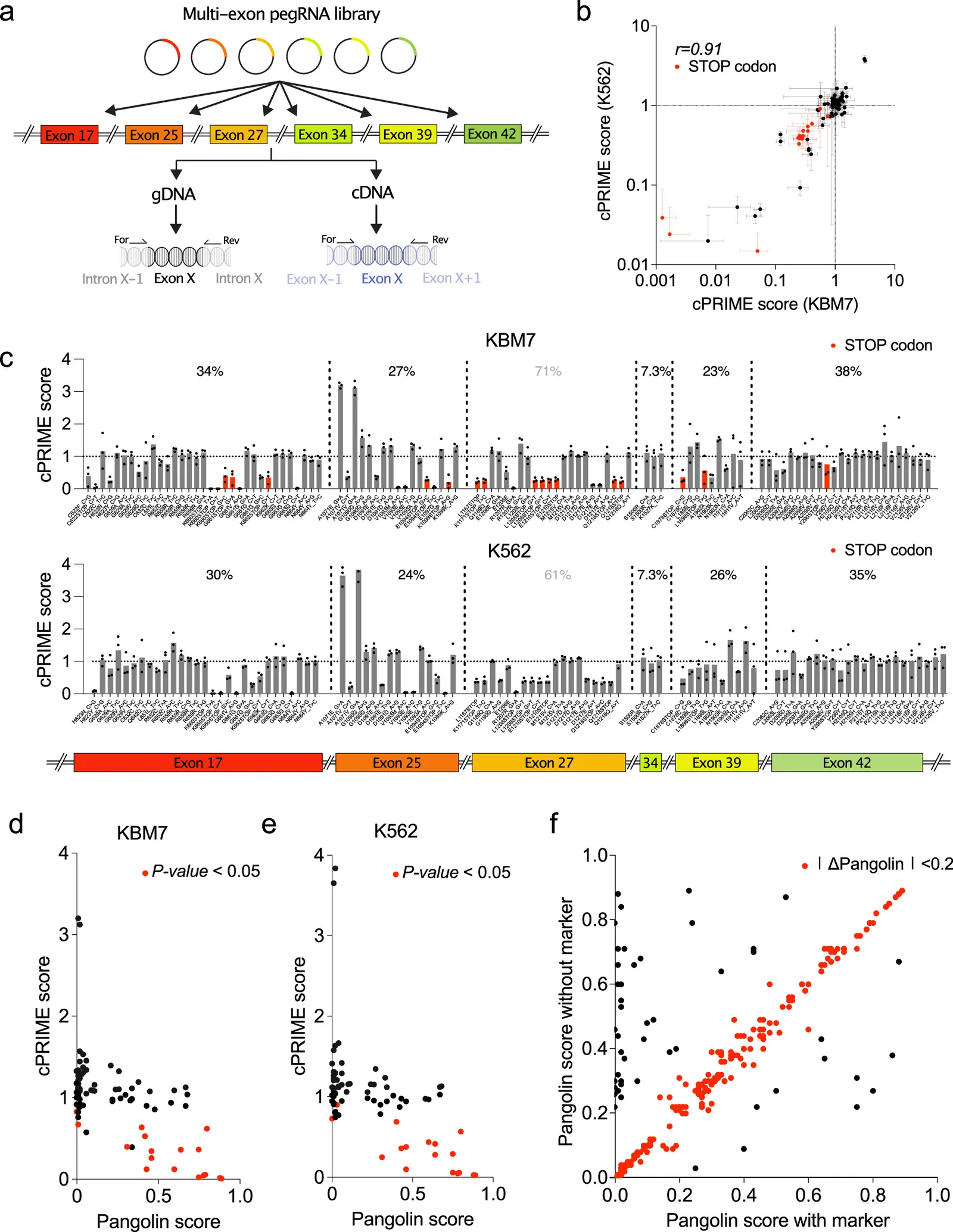
Multi-exon *NF1* screen. **a** Schematic of the *NF1* multi-exon pooled prime editing screen. **b** Correlation of cPRIME scores between KBM7 and K562 cells. Nonsense variants are shown in red. Pearson correlation was used to determine the *r* value. Values represent the mean of biological replicates; error bars, SD (*n* = 3). **c** cPRIME scores in KBM7 (top) and K562 (bottom) cells. Nonsense variants are shown in red. Percentages of variants above threshold (5 RPM) are indicated for each targeted exon. Values represent the mean of biological replicates; *n* = 3. **d** Relationship between Pangolin splice prediction scores and cPRIME scores for variants in KBM7 and **e** K562 cells. Variants with cPRIME scores below 1 and nominal *P-value* < 0.05 are highlighted in red. Nominal significance was estimated in an exploratory analysis by testing the log₂-transformed cPRIME scores against a baseline of 0, corresponding to no change in relative variant RPM between cDNA and gDNA. An unpaired two-stage step-up (Benjamini, Krieger and Yekutieli) t-test was used. Values represent the mean of biological replicates; *n* = 3. **f** Relationship of Pangolin scores between multi-exon screen *NF1* variants with and without marker edits. Marker edits whose predicted Pangolin score is altered by the marker by |ΔPangolin| < 0.2 are shown in red (non-confounding markers). |ΔPangolin| represents the absolute value of the change in Pangolin score. Source data are provided as a Source Data file.

To investigate whether cPRIME can be applied to multiple genomic loci in parallel, we simultaneously targeted *NF1* exons 17, 25, 34, 39 and 42 together with the previously targeted exon 27, using a single pegRNA library (Fig. 3a). The additional exons were selected based on a high density of genomic positions containing predicted splice active variants and ClinVar variants at these positions, while exon 27 was included as an internal reference to assess reproducibility between screens. The final library contained 341 pegRNAs representing 178 individual intended edits across all six exons. These included ClinVar variants as well as additional predicted splice-active variants and nonsense variants (Supp. Fig. 2a–c). Since our previous experiments showed that synonymous marker edits can themselves influence RNA processing, marker sequences for the multi-exon library were evaluated by Pangolin during library design. The pegRNA library was sequenced from the plasmid pool prior to the screens to assess its representation, and showed a narrow distribution with 96.8% of pegRNAs having read counts within one order of magnitude after normalization to the mean pegRNA count (0.24- to 2.4-fold normalized read counts; Supp. Fig. 2d).

Similar to the initial exon 27 screen, cPRIME scores were highly reproducible between KBM7 and K562 cells, with a Pearson correlation coefficient of *r* = 0.91 (Fig. 3b). Furthermore, cPRIME scores for exon 27 variants correlated strongly between the original exon 27 screen and the multi-exon screen, with a Pearson correlation coefficient of r = 0.96, supporting the reproducibility of the approach across pegRNA libraries of different complexity (Supp. Fig. 2f). The fraction of edits detected above threshold ranged from 7.3% for exon 34–38% for exon 42 in KBM7 cells, and from 7.3% for exon 34–35% for exon 42 in K562 cells (Fig. 3c). Exon 27 was excluded from this comparison because some of its pegRNAs were pre-selected from the initial exon 27 screen and therefore do not represent an unbiased estimate of exon-level editing efficiency (Fig. 3c, Supp. Fig. 2e–g).

Nonsense variants consistently showed cPRIME scores below 1, in agreement with reduced RNA abundance caused by nonsense-mediated decay. Interestingly, several variants, most notably, A1071E_G > A (edit: c.3212 C > A, marker: c.3210 G > A) and A1071V_G > A (edit: c.3212 C > T, marker: c.3210 G > A) showed significantly increased cPRIME scores in both cell lines, suggesting increased relative abundance of the edited transcript (Fig. 3c). SNVs may alter local RNA regulatory sequence features, including potential N^6^-Methyladenosine (m6A) methylation-associated motifs which could potentially influence transcript stability or processing. To test this hypothesis, we evaluated the variant sequences with the m6A site predictor deepSRAMP^21^, which predicted an increased probability of an m6A site for both variants relative to wild-type (Supp. Fig. 3). As m6A can either stabilize or destabilize transcripts in a reader- and context-dependent manner, this predicted site represents a candidate mechanism for the altered transcript abundance but does not establish its direction. Therefore, additional experiments such as m6A mapping and RNA-stability assays would be required to illuminate the underlying mechanism.

We next compared experimentally measured cPRIME scores with Pangolin splice prediction scores. Variants with high Pangolin scores showed consistently reduced cPRIME scores, indicating again that predicted splice-altering variants can be detected experimentally by cPRIME (Fig. 3d, e). In contrast, variants with intermediate Pangolin scores showed variable cPRIME scores, ranging from strong to no detectable effect. Most variants with low Pangolin scores showed cPRIME scores around 1, consistent with the absence of predicted splice effects. Variants with cPRIME scores below 1 and nominal *P* < 0.05 were highlighted as candidate hits causing decreased cDNA counts relative to gDNA. These findings indicate that Pangolin and cPRIME provide complementary information. While Pangolin predicts the potential of a variant to alter splicing, cPRIME directly measures the effect of the endogenously introduced variant at the RNA level.

To investigate whether these interpretations are specific to a single splice predictor (Pangolin), we repeated the analysis using the alternative splice predictor SpliceAI^22^. SpliceAI and Pangolin scores were strongly correlated across all analyzed variants (*r* = 0.93; Supp. Fig. 4). These results demonstrate that splice predictions are robust to predictor choice.

Finally, we assessed whether synonymous markers could influence Pangolin splice predictions. During library design, edit-marker combinations were evaluated with Pangolin to identify cases in which the synonymous marker altered the predicted splice gain score relative to the intended edit alone. While those non-neutral markers were included in the library to experimentally assess synonymous marker effects at the RNA level, they were excluded from downstream analyses focused on classifying the effects of intended edits alone (Fig. 3f). This filtering strategy reduces the risk of attributing marker-induced splice effects to the intended edit and supports a more reliable interpretation of cPRIME scores.

### cPRIME distinguishes ClinVar pathogenic *NF1* variants associated with altered RNA

To evaluate whether cPRIME scores can distinguish clinically annotated variant classes, we compared the multi-exon screen results with ClinVar pathogenicity annotations using a receiver operating characteristic (ROC) curve analysis. The analyzed ClinVar variants originated from the screened variant library, which was constructed by first selecting genomic positions containing at least one variant predicted to activate cryptic splice sites and then adding all ClinVar variants found at the same positions. Thus, the ClinVar variants analyzed here are enriched for variants located at possible splice active sites and do not represent an unbiased sample of all ClinVar variants. For this analysis, cPRIME scores were calculated by aggregating across replicates and synonymous marker variants for each intended edit. Variants not passing quality control criteria were excluded (Fig. 3f and Supp. Fig. 5). Variants of uncertain significance, variants with conflicting interpretations, variants lacking ClinVar annotation and nonsense variants were also excluded.

**Fig. 4:**
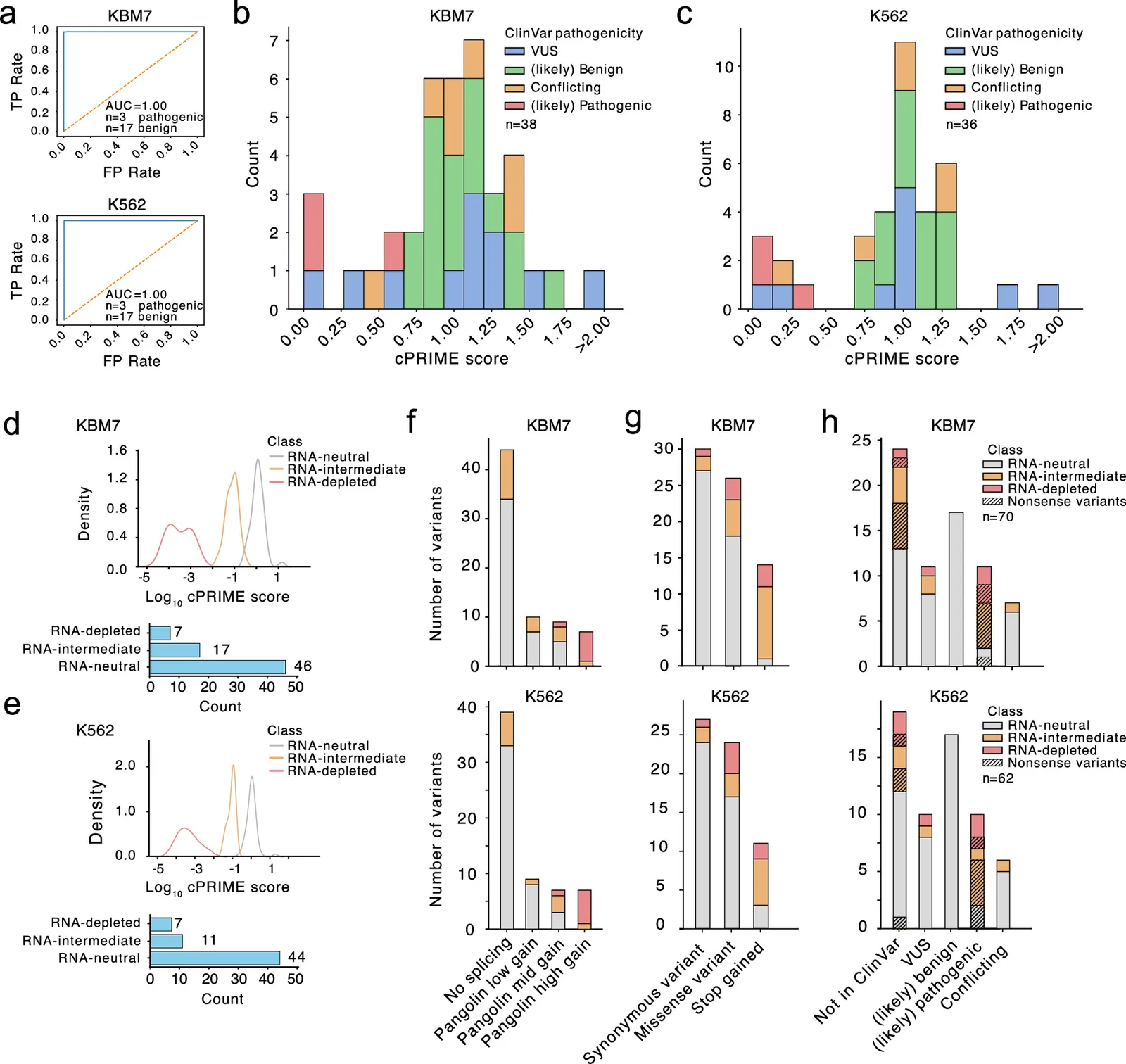
cPRIME distinguishes ClinVar pathogenic *NF1* variants associated with altered RNA abundance. **a** Receiver operating characteristic (ROC) curve analysis assessing the ability of the cPRIME score to distinguish ClinVar-classified pathogenic or likely pathogenic variants from other ClinVar categories in KBM7 (top) and K562 (bottom) cells. Area under the curve (AUC) values are indicated. ROC analysis is based on non-STOP variants only, 3 pathogenic and 17 benign variants in each cell line. **b** Distribution of *NF1* cPRIME scores in KBM7 and **c** K562 cells, grouped by ClinVar pathogenicity classification. Only non-STOP variants listed in ClinVar are shown. **d** Three-component Gaussian mixture model fitted to normalized cPRIME scores in KBM7 and **e** K562 cells. Models were fitted on all variant types, including nonsense variants. Variants were assigned to RNA-depleted, RNA-intermediate and RNA-neutral classes according to the component with the highest posterior probability. Bar plots show the number of variants assigned to each class. **f** Distribution of variants across Pangolin-predicted splice-gain categories in KBM7 (top) and K562 (bottom) cells, grouped by cPRIME response class. **g** Distribution of variant consequence types among cPRIME response classes in KBM7 (top) and K562 (bottom) cells. **h** Distribution of ClinVar pathogenicity classifications among cPRIME response classes in KBM7 (top) and K562 (bottom) cells. Shaded portions within the bars indicate nonsense variants. Counts represent variants included in the *NF1* multi-exon screen. Source data are provided as a Source Data file.

ROC analysis of the pathogenic and benign variants showed that cPRIME scores separated ClinVar pathogenic and likely pathogenic variants from benign and likely benign variants, with an area under the curve (AUC) of 1.0 (3 pathogenic variants and 17 benign variants) in both KBM7 and K562 cells (Fig. 4a). Although this gold-standard set is small, with the complete separation of the 3 pathogenic and 17 benign variants the ROC analysis serves as proof-of-principle that supports the cPRIME-score distributions described below rather than as a definitive performance benchmark. Consistent with this result, pathogenic and likely pathogenic variants showed low cPRIME scores in both cell lines, whereas benign and likely benign variants were predominantly distributed around a cPRIME score of 1 (Fig. 4b, c). Variants of uncertain significance (VUS) spanned a broad range of cPRIME scores, including values close to 1 as well as strongly reduced scores. These findings indicate that cPRIME can provide functional information for clinically annotated *NF1* variants and may help prioritize VUS with potential effects at RNA level.

### Gaussian mixture model to classify *NF1* variants by RNA-level effects

To further classify variant effects in an unbiased manner, we fitted a Gaussian mixture model (GMM) to the log_10_-transformed cPRIME scores. Similar to the ROC analysis, we calculated average cPRIME scores and removed variants not passing quality control. Because the GMM analysis aims to capture experimentally observed groups rather than establish pathogenicity thresholds, nonsense variants were retained in this unsupervised classification. Based on the Bayesian information criterion (BIC) a three-component model was selected for subsequent analyses, reflecting three classes: RNA-neutral, with log_10_-transformed cPRIME scores centered around 0; RNA-intermediate variants, with reduced cPRIME scores; and RNA-depleted, with strongly reduced cPRIME scores (Fig. 4d, e and Supp. Fig. 6, Supp. Data 9). These GMM-derived classes should not be interpreted as ACMG-calibrated thresholds for clinical pathogenicity, but as descriptive categories of variant effects within this screen. In KBM7 cells, the GMM classified 46 variants as RNA-neutral, 17 as RNA-intermediate and 7 as RNA-depleted. In K562 cells, 44 variants were classified as RNA-neutral, 11 as RNA-intermediate and 7 as RNA-depleted.

We then compared these GMM-defined classes with Pangolin splice prediction bins, variant consequence and ClinVar classification. RNA-depleted were predominantly enriched among variants from the Pangolin high-gain bin (score > 0.7), with one additional RNA-depleted assigned to the Pangolin intermediate-gain bin, whereas RNA-neutral mainly consisted of variants with no predicted splice effect or low Pangolin gain scores (Fig. 4f). Similarly, stop-gained variants were preferentially classified as RNA-depleted or RNA-intermediate variants, while synonymous variants were predominantly classified as RNA-neutral (Fig. 4g). Finally, comparison with ClinVar annotations showed that benign and likely benign variants were classified as RNA-neutral, whereas RNA-depleted included pathogenic and likely pathogenic variants as well as variants lacking a ClinVar pathogenicity classification (Fig. 4h). Together, these results indicate that cPRIME scores can identify genetic variants associated with RNA-level alterations.

## Discussion

This study introduces cPRIME, a prime-editing-based framework to measure RNA-level effects of variants introduced at endogenous genomic loci. cPRIME builds on the principle of comparing variant representation between cDNA and genomic DNA, as previously used in saturation genome editing (SGE)^10,11^, but replaces HDR-mediated variant installation with prime editing. By eliminating the need for locus-specific DNA donor template pools required for SGE, cPRIME enables variants across multiple genomic loci to be installed from a single pegRNA library, while - unlike MPRA^6–9^ - preserving their native genomic and RNA-processing context.

The data presented here show that cPRIME detects variant-associated changes at the RNA level across different karyotypes and genomic loci. Reduced cPRIME scores were observed for nonsense variants, consistent with nonsense-mediated decay, and for predicted splice-altering variants, demonstrating the ability of cPRIME to identify variants associated with altered RNA abundance and processing. One broader implication of this study is that synonymous marker edits, which are frequently used in prime-editing screens to improve sensitivity^14–17^, can themselves alter RNA processing. Although such edits preserve the encoded amino acid sequence, they change RNA sequence and can introduce or modify splice-relevant motifs. Synonymous marker edits should therefore not be considered neutral by default. Future pegRNA libraries should incorporate splice-aware computational filtering and, where possible, multiple independent marker designs to distinguish intended variant effects from marker-induced RNA effects.

cPRIME is best positioned as a quantitative screening approach that prioritizes variants with RNA-level effects for mechanistic follow-up. By detecting altered representation of edited variants at RNA level relative to gDNA, cPRIME identifies candidate variants whose effects may arise from altered splicing, nonsense-mediated decay, or other changes in transcript stability. Orthogonal and variant-specific assays, such as isoform-specific RT-PCR, long-read transcript sequencing, or perturbation of RNA decay pathways, can then define the underlying mechanism.

The current implementation of cPRIME relies on targeted amplicon sequencing of matched gDNA and cDNA samples. This targeted readout is an important strength, as it provides a quantitative, sequencing-based measurement of variant representation at both genomic and RNA levels with high sensitivity and moderate sequencing depth. At the same time, it constrains the assay to regions for which suitable gDNA and cDNA amplicons can be designed, and longer transcripts or complex isoform structures may require region-specific amplification strategies or complementary transcript-level assays. Thus, cPRIME enables pooled installation and screening of variants across multiple genomic loci using a single pegRNA library, while the sequencing readout remains target-specific and must be adapted to the genomic and transcript architecture of the region under investigation.

Prime editing efficiency, though steadily improving, remains low and highly variable across target sites and pegRNAs, and this variability determines the coverage required to quantify a given fraction of variants above threshold. In the multi-exon screen, the fraction of variants detected above threshold ranged from 7.3 to 38% in KBM7 cells and 7.3 to 35% in K562 cells (Fig. 3c), despite a screen coverage of 35,000 cells per pegRNA. This reflects the fact that a variant’s representation scales with its editing efficiency relative to library size, meaning the least efficient pegRNAs require the greatest coverage to reach the detection threshold, whereas the same coverage substantially over-samples more efficient pegRNAs. Improved pegRNA design and next-generation prime editors such as PE7^23^ and PE8^24^ are expected to increase editing efficiency while reducing target-site-specific variability, thereby lowering the required screen coverage and enabling increasingly complex cPRIME screens.

Finally, while our ClinVar comparison is limited to a small set of variants, it is directionally consistent with cPRIME capturing RNA-level effects that contribute to variant function. Rather than serving as a universal pathogenicity classifier, cPRIME complements protein-focused functional assays by identifying variants whose effects are mediated through altered RNA abundance or processing. Taken together, cPRIME provides a framework for prioritizing RNA-altering variants and supporting the functional interpretation of genetic variants in cancer and other genetic disorders.

## Methods

### Vector maps

Plasmids and their sequences are deposited at Addgene: PE2_lenti (Addgene #237895) and pPE3 (Addgene #237896).

### Exon 27 pegRNA library design

pegRNAs were designed for exon 27 (UCSC Table Browser: genomic position 29560020 − 29560231) of *NF1* based on the human reference genome hg19. We used the Ensembl transcript ENST00000358273 of the *NF1* gene throughout the manuscript unless stated otherwise. The library contains a total of 98 pegRNAs, of which 50 pegRNAs are based on mutations from the cBioPortal^2^ database (downloaded January 2022).

In addition, for each codon position of exon 27 it was tested whether a nucleotide exchange at one of the three nucleotide positions could theoretically lead to a nonsense mutation. If this was possible and no nonsense mutation has yet been described in cBioPortal at the corresponding nucleotide position, a pegRNA was designed for this position. Thus, we obtained 9 additional pegRNAs for nonsense mutations that were not previously described in patient tumors.

For each codon position in exon 27 for which we designed a pegRNA with an intended edit leading to an amino acid change (50 mutations from the cBioPortal and 9 additional nonsense mutations), a pegRNA with a synonymous mutation as control was designed, referred to as a synonymous edit. For this purpose, the base of the third nucleotide position of the respective codon was changed so that we obtain the same amino acid as in the wild-type. The library contains a total of 39 pegRNAs for synonymous edits. One for each edited codon of exon 27.

As described by Kim et al. as PEER-seq^14^, it is possible to distinguish true edits from sequencing errors at higher sensitivity by an additional synonymous substitution in adjacent codons. For this purpose, we added an additional synonymous substitution, called synonymous marker, to each intended edit (edits with amino acid change as well as synonymous edit). The synonymous marker was positioned at the third nucleotide position of the subsequent codon of an intended edit with an amino acid change or a synonymous edit. If the subsequent codon was an ATG (Met), TGG (Trp), TAA (STOP), TAG (STOP) or TGA (STOP), the synonymous marker was inserted at the third nucleotide position of the codon before the codon with the intended edit. Edits with amino acid changes for which we could not design a synonymous marker were not included in the library.

We used PrimeDesign to generate one pegRNA with a primer binding site (PBS) length of 14 nt and a maximum reverse transcription template (RTT) length of 45 nt for each mutation and its synonymous edit^25^. For the additional nonsense mutations and their synonymous edits, at positions not described in cBioPortal, we had up to 10 pegRNAs generated by PrimeDesign. We selected one of these pegRNAs and added it to our library.

The pegRNAs of the edits for validation were generated manually. For this purpose, the pegRNAs containing an intended edit at the same nucleotide position were used as a template. This intended edit was replaced by an edit for validation, whereby the synonymous marker was retained or removed. In this way, we additionally generated a total of 16 pegRNAs with intended edits for validation. The search sequences for the validation edits were also generated manually. The pegRNA sequences and corresponding library information are provided in Supp. Data 7.

### Design of the multi-exon pegRNA library

Precalculated Pangolin scores for all SNVs in the *NF1* gene were downloaded from Zenodo (10.5281/zenodo.15649337) and annotated with ClinVar classifications (downloaded September 2025). Following Zeng et al.^20^ SNVs were binned in low gain (score >0.2), mid gain (score >0.4) and high gain (score >0.7), as well as low loss (score <−0.2), mid loss (score <−0.4) and high loss (score <−0.7).

*NF1* gene exons 17, 25, 34, 39, and 42 (transcript ENST00000358273) were chosen based on the highest density of predicted splice active variants (by Pangolin) with at least a low splice gain score and variants listed in ClinVar at these positions that were analyzable with amplicon sequencing. These were distributed across the whole *NF1* gene. Exon 27 was also included in this screen as a reference.

A set of splice active variants was extracted from this data. We reasoned that variants which activate cryptic splice sites should show a strong depletion in cDNA levels. Thus, we selected all variants which showed at least a low gain in splice activity and where the splice loss score as predicted by Pangolin was smaller than the splice gain score and where the splice effect was located at least 10 base pairs apart from the next canonical splice site. In addition, variants listed in ClinVar were included if they occurred at the same genomic coordinates where Pangolin identified splice gain variants. Five additional nonsense variants for each exon were computed that do not themselves impact splicing. Following the previous screen, for each nonsense variant an additional synonymous variant at the codon position was designed as a control. Additionally, we added five pegRNAs encoding nonsense variants and four pegRNAs with low cPRIME scores from the previous exon 27 screen as controls.

For each variant, at least one synonymous marker was designed in the flanking codons while minimizing changes to the splicing properties of the intended edit. This was assessed by calculating Pangolin scores for the combined intended edit plus synonymous marker variant, and then comparing the predicted splice-site score and position to those of the intended edit alone. Where available, two of these marker variants were designed for the splice active variants. Additionally, to test the potential risk of false positive or false negative hits one confounding synonymous marker variant was designed that alters the predicted splicing gain from Pangolin of the intended edit by at least 0.2.

Following the previous exon 27 screen, we designed pegRNAs using PrimeDesign^25^. According to Yu et al.^26^ and our previous findings, we kept pegRNAs with fewer than five consecutive thymidines, without BsmBI recognition sequences (CGTCTC and GAGACG), and with an RTT length between 12 and 30 nucleotides. Designs with a PBS length of 12 were prioritized if the RTT was longer than 12 nucleotides and 11 otherwise. Finally, remaining pegRNAs for each variant were sorted to select the most compact pegRNA design.

The final library contains 341 pegRNAs from 178 individual intended edits across 6 exons. The individual edits consist of 74 variants which are unknown in ClinVar, 32 which are VUS, 13 have conflicting classifications of pathogenicity and 59 have a pathogenicity class assigned to it. A total of 98 intended edits do not show a predicted splicing effect while 41, 24 and 15 exhibit a low, medium or high predicted splicing effect by Pangolin, respectively. The library contains a total of 36 nonsense variants, 78 missense variants and 64 synonymous variants (Supp. Fig. 2a–c). The pegRNA sequences and corresponding library information for the multi-exon library, as well as the primer sequences used for amplification of the individual exons from gDNA and cDNA, are provided in Supp. Data 8.

### K562, KBM7 and Lenti-X 293T cell culture

K562 (ATCC, CCL-243) and KBM7 (Horizon Inspired Cell Solutions, C628) cells were cultured in complete RPMI (Thermo Fisher Scientific, 11875093) supplemented with 10% fetal bovine serum and 1% antibiotics (pen/strep). Lenti-X 293 T cells (Takara, 632180) were cultured in complete DMEM (Thermo Fisher Scientific, 11995073) supplemented with 10% fetal bovine serum and 1% antibiotics (pen/strep).

### Generating stable prime editor cell lines

The Cas9 (H840A) and RT fragment of pCMV-PE2 vector (Addgene plasmid #132775) were amplified (with primers 5‘-ttcaggtgtcgtgacgtacggccaccatgaaacggacagc-3’ and 5‘-ctctccactgccgctagGgactttcctcttcttcttgggctc-3’). EF1a-CasRx-2A-EGFP (Addgene plasmid #109049) was digested with BsiWI and NheI. NEBuilder HiFi DNA Assembly Master Mix (NEB, E2621L) was used to clone fragment Cas9 (H840A) and RT into the EF1a-CasRx-2A-EGFP backbone, which resulted in vector PE2_lenti (lentiviral prime editing backbone).

Lentivirus of PE2_lenti was produced according to the protocol below. K562 and KBM7 cell lines were transduced with lentivirus PE2_lenti, and GFP-positive populations were sorted with BD FACSMelody^TM^ Cell Sorter, resulting in KBM7_PE and K562_PE cell lines. For all the following experiments the KBM7_PE population was used while for K562_PE cells a single clone was grown and used.

### Generating MLH1 knock-out cell lines

MLH1 knock-out clones were generated by electroporation of Cas9:gRNA ribonucleoprotein (RNP) complex using the Neon NxT (Invitrogen). Therefore, a predesigned Alt-R™ CRISPR-Cas9 crRNA against MLH1 (Sequence: CTTCACTGAGTAGTTTGCAT, HS.Cas9.MLH1.1.AG, IDT) was mixed in equimolar concentrations with Alt-R™ CRISPR-Cas9 trRNA (IDT, 1072532), followed by heating at 95 °C for 5 min and cooling afterward to form crRNA:tracrRNA duplexes. RNP complex was formed by adding Alt-R™ S.p. Cas9-RFP V3 (IDT, 10008162) in a 1:1.2 ratio with crRNA:tracrRNA duplex and incubating for 20 min. 200,000 cells in 10 ul of K562_PE or KBM7_PE were used for the electroporation with 1 ul RNP. For K562_PE cells, electroporation was carried out at 1700 V, 20 ms, 1 pulse, for the KBM7_PE cells at 1600 V, 10 ms, 3 pulses. Successful electroporation was confirmed by checking the RFP signal via FACS measurement on BD FACSMelody^TM^ Cell Sorter. Cell populations were grown and expanded for 14 days following FACS sorting into single clones. Successful knock-out of MLH1 in grown single clones was confirmed by sequencing the genomic target site of MLH1. The K562_PE_MLH1 and KBM7_PE_MLH1 cell lines generated in this study are available from the corresponding author upon reasonable request.

### Cloning pegRNA expression plasmid

BsmBI restriction site was removed by site directed mutagenesis PCR of vector sgLenti (Addgene plasmid #105996) resulting in MP783∆BsmBI. The pPapi expression cassette (u6-stuffer-H1) was amplified from vector pPapi (Addgene plasmid #96921) and cloned with NEBuilder HiFi DNA Assembly Master Mix according to the manual (NEB, E2621L) into MP783∆BsmBI, resulting in the pPE3 plasmid.

### Cloning of pegRNA libraries

For cloning the pegRNA libraries, the designed pegRNA sequences (length 248-284nt) were synthesized as an Oligo Pool from Twist Bioscience. The structure is 5‘-GTTGATCCGTCTCAACCG-(N)_20peg_-GTTTTAGAGCTAGAAATAGCAAGTTAAAATAAGGCTAGTCCGTTATCAACTTGAAAAAGTGGGACCGAGTCGGTCC-(N)_25-60_-TTTTTTTTGAACAGACGCAGGTGAACCGACGGATGATCTCGTGCACCGGTTCAAAAAAAACACCTGCGATCAAAC-(N)_20nick_-CGGGAGAGACGGTTCC-3’. (N)_20peg_ represents the different pegRNA-Spacer, (N)_25-60_ the pegRNA RT-PBS and repair template of variable length, and (N)_20nick_ represents the nickRNA-Spacer. The nickRNA-Spacer was included in the exon 27 library for possible usage of the library as PE3 after insertion of the nickRNA-TRACR.

The template pool was PCR-amplified using Phusion Flash High-Fidelity PCR Master Mix (Thermo Fisher Scientific, F548L) according to the manufacturer’s protocol with 6 ng pegRNA template DNA, 1 uM forward (5‘-ACTGGCTATTAAGTTGATCCGTCTCAACCG-3‘) and reverse primer (5’- TAGCCTAGTTATGGAACCGTCTCTCCCG-3’). The PCR was performed with 1x (98 °C − 3 min), 12x (98 °C − 10 s, 60 °C − 20 s, 72 °C − 30 s) and 1x (72 °C − 3 min). PCR product was purified with NucleoSpin Gel and PCR Clean-up (Macherey-Nagel, 740609.250). The backbone vector pPE3 was prepared by digestion with BsmBI-v2 (NEB, R0739L) at 55 °C for 4 h, followed by 1% agarose gel separation and gel extraction via NucleoSpin Gel and PCR Clean-up (Macherey-Nagel, 740609.250). Golden Gate Assembly was used to assemble 75 ng BsmBI-v2 digested pPE3 with 5 ng of amplified pegRNA library insert (2:1 ratio) in a total volume of 20 ul, containing 1 ul BsmBI-v2 (10 U/ul), 1 ul T4 DNA ligase (5 U/ul, Thermo Fisher Scientific, EL0011) and 1x T4 DNA ligase buffer at 42 °C for 1 h. A fraction of 2 ul of the Golden Gate Assembly was transformed into 25 ul electrocompetent Endura cells (Lucigen, 60242-2). Endura cells were recovered, cultured overnight in 200 ml LB (100 ug/ml Carbenicillin) and used for Midiprep (NucleoBond Xtra Midi kit for transfection-grade plasmid DNA, Macherey-Nagel, 740410.50). In parallel, a fraction of the transformed cells was plated to determine the total number of transformed clones. The resulting coverage was 1600x clones per pegRNA for the exon 27 library and 13,000x for the multi-exon library, which ensures even representation of all library pegRNAs and their narrow distribution. The distribution of pegRNA sequences was analyzed by amplifying the pegRNA cassette of the plasmid pool. Therefore, the Phusion Flash High-Fidelity PCR Master Mix (Thermo Fisher Scientific, F548L) was used according to the manufacturer’s protocol with 350 ng plasmid DNA, 0.2 uM forward (5‘-AATGATACGGCGACCACCGAGATCTACAC-(N)_8_-ACACTCTTTCCCTACACGACGCTCTTCCGATCTatcttgtggaaaggacgaaacaccG-3‘) and reverse primer (5’-CAAGCAGAAGACGGCATACGAGAT-(N)_8_-GTGACTGGAGTTCAGACGTGTGCTCTTCCGATCTGCACGAGATCATCCGTCGGTTC-3’) with (N)_8_ being an 8 nt index for sequencing on the Illumina sequencing platform. The PCR was performed with 1x (98 °C − 3 min), 18x (98 °C − 10 s, 72 °C − 40 s) and 1x (72 °C − 5 min). The PCR product was purified with NucleoSpin Gel and PCR Clean-up, followed by next-generation sequencing. The NGS results showed a narrow distribution of pegRNA sequences, with read counts for all pegRNA sequences falling within a single order of magnitude (see Fig. 1e and Supp. Fig. 2d, Supp. Data 4).

### Cloning of exon 27 validation pegRNAs

For cloning single pegRNAs, all sequences were synthesized as one pool from IDT with 50 pmol of each pegRNA. The pool was dissolved in H_2_O to obtain 1 uM. 0.5 ul was used for the following amplification step. PCR amplification and Golden Gate Assembly were performed identically to the exon 27 library. The assembled products were transformed into One Shot™ Stbl3™ Chemically competent *E. coli* cells (Thermo Fisher Scientific, C737303). The obtained plasmids were analyzed by Sanger sequencing (Genewiz).

### Lentivirus production for screens and validation experiments

Lenti-X 293 T cells were seeded at 70,000 cells per cm2 in 15 cm dishes with 30 ml medium (DMEM, 10% FBS) and incubated overnight at 37 °C, 5% CO2. The next morning, 15 ug pegRNA library plasmid, 6 ug psPAX2 (Addgene #12260), 6 ug pMD2.G (Addgene #12259) and 5250 ul DMEM (wo FBS) were mixed with 108 ul Turbofect Transfection reagent (Thermo Fisher Scientific, R0532), vortexed, incubated for 20 min at RT and added to the cells. 48 h and 72 h post-transfection, supernatant was harvested, passed through 0.45 um filters (Millipore, Stericup), and aliquots were stored at −80 °C. For validation/single pegRNA experiments, this setup was downscaled.

### Prime editing screens

For the exon 27 screen, KBM7_PE_MLH1 cells were transduced with lentivirally packaged exon 27 library at a multiplicity of infection (MOI) = 0.7 and 100,000x cell coverage as triplicates. Cells were cultured in IMDM (Thermo Fisher Scientific, 12440061) with 10% FBS and 1x Anti-Anti (Thermo Fisher Scientific, 15240062) in a 37 °C incubator with 5% CO_2_. 48 h after transduction, puromycin (2 ug/ml) was added for selection and used for the whole screening time. Coverage at the cell level was kept above 30,000x throughout the entire screen, and the culture was diluted with fresh medium when cell density reached 2 million/ml. 21 days post transduction, aliquots of 1 million cells (≙10,000x coverage) for each triplicate were frozen.

K562_PE_MLH1 cells were transduced with lentivirally packaged exon 27 library at a multiplicity of infection (MOI) = 0.55 and 23,000x cell coverage as triplicates. Cells were cultured in RPMI (Thermo Fisher Scientific, 11875093) with 10% FBS and 1x Anti-Anti (Thermo Fisher Scientific, 15240062) in a 37 °C incubator with 5% CO_2_. 48 h after transduction, puromycin (2 ug/ml) was added for selection and used for the whole screening time. Coverage at the cell level was kept above 30,000x throughout the entire screen, and the culture was diluted with fresh medium when cell density reached 1 million/ml. 36 d after transduction, double positive populations (GFP, mCherry) were sorted for each triplicate (40,000x cell coverage). 45 days post transduction, aliquots of 1 million cells (≙10,000x coverage) for each triplicate were frozen. For the multi-exon screen, KBM7 and K562 cells were transduced at an MOI of 0.3 at a coverage of 35,000x. The coverage was maintained throughout the screen. At 45 days post-transduction, aliquots of 12,000,000 cells were harvested per cell line per replicate, and the cell pellets were frozen.

### Arrayed validation experiments

KBM7_PE_MLH1 cells were transduced with lentivirally packaged single pegRNAs selected for validation experiments. Cells were cultured in IMDM (Thermo Fisher Scientific, 12440061) with 10% FBS and 1x Anti-Anti (Thermo Fisher Scientific, 15240062) in a 37 °C incubator with 5% CO_2_. 48 h after transduction, puromycin (2 ug/ml) was added for selection and used for the whole editing time. 24/21 days post transduction, aliquots of 150,000 cells were frozen in triplicate.

K562_PE_MLH1 cells were transduced with lentivirally packaged single pegRNAs selected for validation experiments. Cells were cultured in RPMI (Thermo Fisher Scientific, 11875093) with 10% FBS and 1x Anti-Anti (Thermo Fisher Scientific, 15240062) in a 37 °C incubator with 5% CO_2_. 48 h after transduction, puromycin (2 ug/ml) was added for selection and used for the whole editing time. After 31/21 d of editing/post-transduction, aliquots of 150,000 cells were frozen in triplicate.

### Genomic DNA (gDNA) extraction

Cell pellets from the exon 27 screen and validation experiments were thawed, and gDNA was extracted using DNeasy Blood & Tissue Kit (Qiagen, 69504) according to the manual. DNA was eluted in 80/50 ul for screen/validation samples, and concentration was measured with NanoDrop One (Thermo Fisher Scientific).

For the multi-exon screen, the pellets were thawed, and the genomic DNA was extracted using phenol-chloroform. The samples were resuspended in 5 ml of P1 buffer (Qiagen, 19051) containing 100 µg/ml RNase A (Sigma-Aldrich, 10109142001) and 0.5% SDS (Sigma-Aldrich, 71725-50 G), followed by incubation at 37 °C for 30 min. Then, Proteinase K (Sigma-Aldrich, 70663-4) was added (final concentration 100 µg/ml) and incubated at 55 °C for 30 min. After digestion, the samples were homogenized by passing through an 18 G needle three times and then through a 22 G needle three times. The homogenized samples were mixed with 5 ml Phenol:Chloroform:Isoamyl alcohol (Thermo Fisher Scientific, 15593031) and thoroughly mixed. The samples were then centrifuged at room temperature (RT) for 5 min at 1500 xg. The aqueous phase was transferred to ultracentrifuge tubes and thoroughly mixed with 0.5 ml of 3 M sodium acetate (Sigma-Aldrich, S2889-250G) plus 4 ml of isopropanol (Sigma-Aldrich, 650447-1 L) at RT before centrifugation at 15,000 xg for 15 min. The gDNA pellets were gently washed with 5 ml of 70% ethanol (Thermo Fisher Scientific, 17740239) and dried at 37 °C. The pellets were resuspended in H2O, and the gDNA concentration was adjusted to 1 µg/µl. The degree of gDNA fragmentation was assessed on a 1% agarose gel.

### Amplification of target *NF1* exons from genomic DNA

For amplifying the genomic target site of the exon 27 screen samples, the harvested gDNA from a 10,000x cell coverage was used for each triplicate. For the first round of two nested PCRs, the total volume was 50 ul containing 12 ug gDNA, 0.2 uM forward (5’ -ACCTAGAGAACTGGCATGTAAGAGAAGC-3’) and reverse primer (5’-TCACCTAACTAAACATCTTTCTTCTGGCTCTG-3’), 200 uM dNTP, 1x Titanium Taq PCR buffer and 1 ul Titanium Taq (Takara Bio, 639209). PCR cycles were 1x (95 °C − 5 min), 16x (95 °C − 30 s, 55 °C − 30 s, 68 °C − 30 s), 1x (68 °C − 3 min). Two first-round PCR reactions for each triplicate were pooled, and a fraction was used as template for the second-round PCR. The total volume of the second-round PCR was 50 ul containing 2 ul pooled first-round PCR, 0.2 uM forward (5’- AATGATACGGCGACCACCGAGATCTACAC -(N)_8_-ACACTCTTTCCCTACACGACGCTCTTCCGATCTGCATCTGTTTGTCCACATTAGGC-3’) and reverse primer (5’-CAAGCAGAAGACGGCATACGAGAT-(N)_8_- GTGACTGGAGTTCAGACGTGTGCTCTTCCGATCTTCCCCGCTTACTCTAATCACTTAC-3’) with (N)_8_ being an 8 nt index for sequencing on the Illumina NovaSeq platform, 200 uM dNTP, 1x Titanium Taq PCR buffer and 1 ul Titanium Taq (Takara Bio, 639209). PCR cycles were 1x (95 °C − 5 min), 14x (95 °C − 30 s, 53 °C − 30 s, 68 °C − 30 s), 1x (68 °C − 3 min). The resulting PCR product (393 bp) was extracted from a 2% agarose gel.

For amplifying the genomic target site of the validation (single pegRNA) samples conditions were slightly modified: only 1 ug harvested gDNA was used for each triplicate, cycles of the first-round PCR varied from 16-18 cycles and the cycles for the second-round PCR varied from 15-16 cycles.

For amplifying the genomic target site of the multi-exon screen samples, the harvested gDNA from a 35,000x cell coverage was used for each triplicate. For the first round of two nested PCRs, the total volume was 100 ul containing 50 ug gDNA, 0.2 uM forward and reverse external primers that bind inside the neighboring intronic regions of each exon (Supp. Data 8), 200 uM dNTP, 1x Titanium Taq PCR buffer and 1.5 ul Titanium Taq (Takara Bio, 639209). PCR cycles were 1x (95 °C − 5 min), 16x (95 °C − 30 s, 55 °C − 30 s, 68 °C − 30 s), 1x (68 °C − 3 min). The first-round PCR reactions for each triplicate were pooled, and a fraction was used as template for the second-round PCR as before using exon-specific Illumina primers (Supp. Data 8).

### RNA extraction and cDNA synthesis

Cell pellets (exon 27 screen 1 million cells, validation 150,000 cells, multi-exon screen 12 million per sample) were thawed, and RNA was extracted using the RNeasy Mini Kit (Qiagen, 74106) according to the manual. RNA was eluted in 35 ul H_2_O for the exon 27 screen, and 50 µL for the multi-exon screen, and concentration was measured with NanoDrop One (Thermo Fisher Scientific). RevertAid H minus first strand cDNA synthesis kit (Thermo Fisher Scientific, K1632) was used for the following cDNA synthesis according to the manual using oligo (dT)_18_ primer. The reaction was performed for 60 min at 42 °C and afterward terminated by 5 min at 70 °C.

### Amplification of *NF1* exons from cDNA

For amplifying the cDNA target site of the screen samples a fraction (10-25% of total RNA was used for cDNA) of the total synthesized cDNA from the full cell coverage of each screen was used for each triplicate. For the exon 27 screen samples, the total volume of the PCR was 50 ul containing 20 ul cDNA, 0.4 uM forward (5’- AATGATACGGCGACCACCGAGATCTACAC-(N)_8_ -ACACTCTTTCCCTACACGACGCTCTTCCGATCTGGTCTCATGCACTCCATAGGC-3’) and reverse primer (5’- CAAGCAGAAGACGGCATACGAGAT-(N)_8_ - GTGACTGGAGTTCAGACGTGTGCTCTTCCGATCTCCAGAACTCGAGCTAGTTCATC −3’) with (N)_8_ being an 8 nt index for sequencing on the Illumina NovaSeq platform, 200 uM dNTP, 1x Titanium Taq PCR buffer and 1 ul Titanium Taq (Takara Bio, 639209). PCR cycles were 1x (95 °C − 5 min), 28x (95 °C − 30 s, 55 °C − 30 s, 68 °C − 30 s), 1x (68 °C − 3 min). For the multi-exon screen, the PCR reaction volume was increased to 100 µL using the primers listed in Supp. Data 8. The PCR protocol was kept the same as for the exon 27 screen. The resulting PCR products were extracted from a 2% agarose gel.

For amplifying the RNA/cDNA target site of the validation (single pegRNA) samples conditions were slightly modified: (30% of total RNA was used for cDNA) the cycles of the PCR varied from 30–32 cycles.

### Sequencing and data analysis

All amplified gDNA and cDNA samples were sent to Novogene for NGS. Paired-end sequencing was performed using MiniSeq, NovaSeq 6000 and NovaSeq X Plus.

The distribution of pegRNAs in the library plasmid pool was analyzed via MAGeCK (version 0.5.9.5)^27^.

FASTQ files that ran on different lanes during sequencing were concatenated, and FLASH (version 1.2.11) was used to merge overlapping reads from paired-end sequencing to represent the entire length of the analyzed exons^28^. To exclude false-positives due to sequencing errors, a quality threshold was defined so that only matches were counted if each nucleotide within the search sequence region had an Illumina quality score of at least 37 (ASCII: F). To do this, all nucleotides in the FASTQ files that did not meet the quality cut-off were masked with an X.

In-house scripts were used to determine read counts for each variant. Read counts were determined by counting the occurrence of each intended edit in combination with its synonymous marker variant. For this, unique search sequences were generated for each variant that contain the intended edit together with its synonymous marker variant and flanking 10 bp of the reference genome sequence. Scripts to generate the search sequences were written in R (version 4.3.0) and Python (version 3.12.9). The cPRIME score was then calculated from the read counts using the following formula:

1 $${{{\rm{cPRIME}}}}\,{{{\rm{score}}}}=\frac{{{{\rm{Read}}}}\,{{{\rm{counts}}}}\,{{{\rm{cDNA}}}}({{{\rm{RPM}}}})}{{{{\rm{Read}}}}\,{{{\rm{counts}}}}\,{{{\rm{gDNA}}}}({{{\rm{RPM}}}})}$$

For the multi-exon screen, scripts were refined for increased usability and speed. In particular, the masking algorithm uses binary batch processing, and the counting script makes use of the Aho-Corasick algorithm^29^. Briefly, the Aho-Corasick algorithm constructs a finite state machine from a prefix tree once and uses it to simultaneously match all search sequences in the reads. Using failure links, the resulting automaton scans each read in linear time and reports all matching search sequences in a single pass.

The initial analysis pipeline was implemented in Bash (version 3.2.57) and Python (version 3.11.3). The advanced analysis pipeline was implemented in Bash (version 5.0.17) and Python (version 3.12.9).

To show that we can distinguish true prime-edit reads from sequencing errors in next-generation sequencing by using the synonymous marker, we calculated the odds ratio (signal-to-background ratio) using Fisher’s exact test between reads from cells at day 45 for K562 or cells at day 21 for KBM7 and reads from unedited cells in the initial exon 27 screen using the following formula:

2 $${{{\rm{Odds}}}}\,{{{\rm{Ratio}}}}=\frac{\frac{{{{\rm{Reads}}}}\,{{{\rm{of}}}}\,{{{\rm{SNV}}}}\,{{{\rm{at}}}}\,{{{\rm{day}}}}\,21/{{{\rm{day}}}}45+1}{{{{\rm{Reads}}}}\,{{{\rm{of}}}}\,{{{\rm{WT}}}}\,{{{\rm{sequence}}}}\,{{{\rm{at}}}}\,{{{\rm{day}}}}21/{{{\rm{day}}}}45+1}}{\frac{{{{\rm{Reads}}}}\,{{{\rm{of}}}}\,{{{\rm{SNV}}}}\,{{{\rm{in}}}}\,{{{\rm{unedited}}}}\,{{{\rm{cells}}}}+1}{{{{\rm{Reads}}}}\,{{{\rm{of}}}}\,{{{\rm{WT}}}}\,{{{\rm{sequence}}}}\,{{{\rm{in}}}}\,{{{\rm{unedited}}}}\,{{{\rm{cells}}}}+1}}$$

An Overview of all odds ratios can be found in Supp. Data 3.

### SNV class assignment and ROC analyses

RPM values per variant with marker edit were averaged across replicates. Next, variants that harbor synonymous marker edits that substantially alter the splicing properties of the intended edit were removed. The RPM scores of the remaining variants were used to calculate cPRIME scores across synonymous marker variants to remove the potential bias for one intended edit to contribute multiple times to the model. Additionally, we log-transformed the data to make it symmetric around 0.

cPRIME scores were used for ROC analysis to assess their ability to predict ClinVar pathogenicity classification. Here, we grouped variants in pathogenic (including variants classified as pathogenic and likely pathogenic in ClinVar) and benign (including variants classified as benign and likely benign in ClinVar). Variants annotated as uncertain significance, with conflicting interpretations or lacking ClinVar annotation, were excluded from this analysis. Nonsense variants were also excluded since their pathogenicity is usually well established, which would inflate performance. ROC curves and area under the curve (AUC) values were computed in Python (version 3.12.9) using the sklearn.metrics.roc_curve and sklearn.metrics.auc functions. ClinVar annotations were downloaded in September 2025.

### Unsupervised Gaussian mixture model-based variant classification

A three-component Gaussian mixture model (GMM) was fitted to the cPRIME scores to classify variants in RNA-depleted, RNA-intermediate and RNA-neutral. Variants were assigned to the components based on their maximum posterior probability. The number of components was chosen using the Bayesian information criterion (BIC) (Supp. Fig. 6). The KBM7 cell line showed the best score with three components (BIC = 179.61). A four-component model yielded the lowest BIC in K562. Comparison of variant classification between three- and four-component models in K562 showed high concordance, with 60 of 62 variants assigned to equal classes. The additional component in the four-component model captured a single outlier variant with a high cPRIME score, rather than defining a broadly applicable class. Considering the statistical support in KBM7, the high concordance between three- and four-component models in K562, and the biological interpretability of three transcript-response categories (RNA-depleted, RNA-intermediate and RNA-neutral), we selected the model with three components for subsequent analyses.

To reduce the influence of outliers during fitting, log-transformed cPRIME scores were clipped at −4 and 4 before modeling. This step prevents single extreme values from dominating component estimation, which is a known limitation of GMMs. Clipping was applied for all data points equally and did not affect the relative ordering of most scores. Specifically, only 1 variant and 3 variants in K562 and KBM7 cell lines fell outside our predefined threshold. We confirmed that the overall model structure was stable after refitting the model on unclipped data, with differences mainly affecting component boundaries due to the influence of extreme values during model fitting. Models were fitted in Python (version 3.12.9) using the sklearn.mixture.GaussianMixture function with the default covariance structure (covariance_structure=’full’) such that each component can have its own variance.

### Splice site prediction with Pangolin and SpliceAI

In the exon 27 screen, splice site usage was predicted with Pangolin (version 1.0.2) using the GRCh37 reference genome along with the liftover gencode annotations provided by the original authors of Pangolin^20^. Since the multi-exon screen was designed on the GRCh38 reference genome, the liftover was not necessary for this second screen. All scores were calculated on the combined variant and synonymous marker substitution unless stated otherwise. An overview of all predicted Pangolin scores can be found in Supp. Data 5 and 8.

SpliceAI^22^ (version 1.3.1) predictions were run on the multi-exon screen combined variants and can be found in Supp. Data 8 as well.

### m6A site prediction with deepSRAMP

Potential effects of variants on N^6^-Methyladenosine (m6A) modification were assessed using the deep learning-based m6A predictor deepSRAMP^21^. To identify variant-associated changes in predicted m6A modification probability, we ran the online prediction tool (http://www.cuilab.cn/deepsramp/; accessed June 2026) on both the reference sequence and the combined intended edit with synonymous marker variants. Each input was augmented with 200 nucleotides of mature RNA sequence context on both sides of the combined edit.

### Statistics & reproducibility

For all experiments, the number of technical and/or biological replicates is listed in the Figure legends or text. No data were excluded from the analyses. The experiments were not randomized. The Investigators were not blinded to allocation during experiments and outcome assessment. Unless otherwise indicated, statistical significance was calculated using a t-test. Pearson correlation was used to determine the r values. Statistical analyses were performed using GraphPad Prism 10 (GraphPad Software) or the R language and Python programming environments.

For the odds ratio analysis (Supp. Data 3), GraphPad Prism 10 and Python (version 3.12.9) were used to create plots. One-way ANOVA followed by Dunnett’s multiple comparisons test was performed using GraphPad Prism 10. Significance levels were determined using a ratio paired t-test and an unpaired two-tailed t-test in Prism 10, unless otherwise stated.

### Reporting summary

Further information on research design is available in the Nature Portfolio Reporting Summary linked to this article.

## Supplementary information

Supplementary Information

Description of Additional Supplementary Files

Supplementary Data 1-9

Reporting Summary

Transparent Peer Review file

## Source data

Source Data 1

Source Data 2

## Data Availability

FASTQ files generated in this study have been deposited in the SRA database under accession code: PRJNA1257064. Plasmids and their sequences are deposited at Addgene: PE2_lenti (Addgene #237895) and pPE3 (Addgene #237896). Source data are provided with this paper.

## Code availability

Scripts for PrimeDesign input, searching sequences, as well as Python and Bash scripts to count edits, can be found on GitHub: https://github.com/Boettcher-Lab/cPRIME. The scripts are also available at Zenodo under the following https://doi.org/10.5281/zenodo.21991157. The code is available under the MIT License.
