# Supplementary material for: cPRIME enables endogenous mapping of genetic variant effects at RNA level
Source: Nat Commun. 2026 Oct 15;17:10872. doi: 10.1038/s41467-026-78344-9 (PMC13675207; doi:10.1038/s41467-026-78344-9)
Supplement: Supplementary file 1 [file 41467_2026_78344_MOESM1_ESM.pdf]

cPRIME enables endogenous mapping of genetic variant effects at RNA level

Ghanem El Kassem[1*], Constanze Westphal[1*], Marvin Doebel[1*], Jasmine Hillmer[1*], Tanja Seifert[1], Michael Boettcher[1#]

[1] Institute of Molecular Medicine, Section for Molecular Medicine of Signal Transduction, Faculty of Medicine, Martin Luther University Halle-Wittenberg, 06120 Halle, Germany

[#]Correspondence to: michael.boettcher@medizin.uni-halle.de

*equal contribution

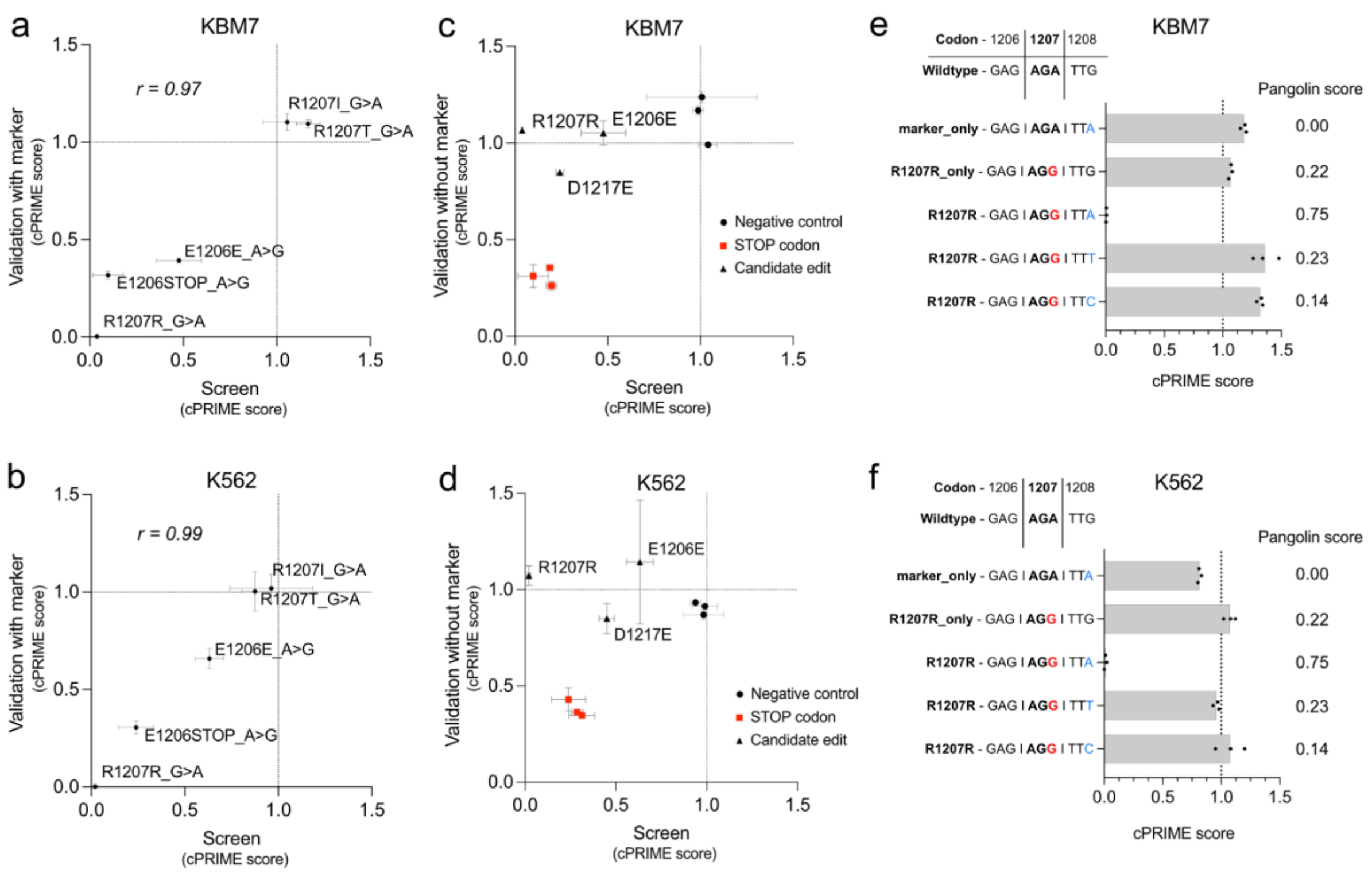

Supplementary Figure 1 - Synonymous marker edits can influence RNA processing. a, Technical validation of selected variants with synonymous marker edits from pooled prime editing screens in KBM7 and **b,** K562 cells. Values represent the mean of biological replicates; n=3, r = Pearson correlation coefficient. **c,** Comparison of the effects of selected variants without synonymous marker edits to prime editing screen results (with synonymous marker edits) in KBM7 and **d,** K562 cells. Nonsense (STOP) variants are shown in red. Values represent the mean of biological replicates; n=3. **e,** Effects of different codon 1208 marker edits on cPRIME score in KBM7 and **f,** K562 cells. Values represent the mean of biological replicates; n=3. The Pangolin score for each variant-marker combination is displayed. Source data are provided as a Source Data file.

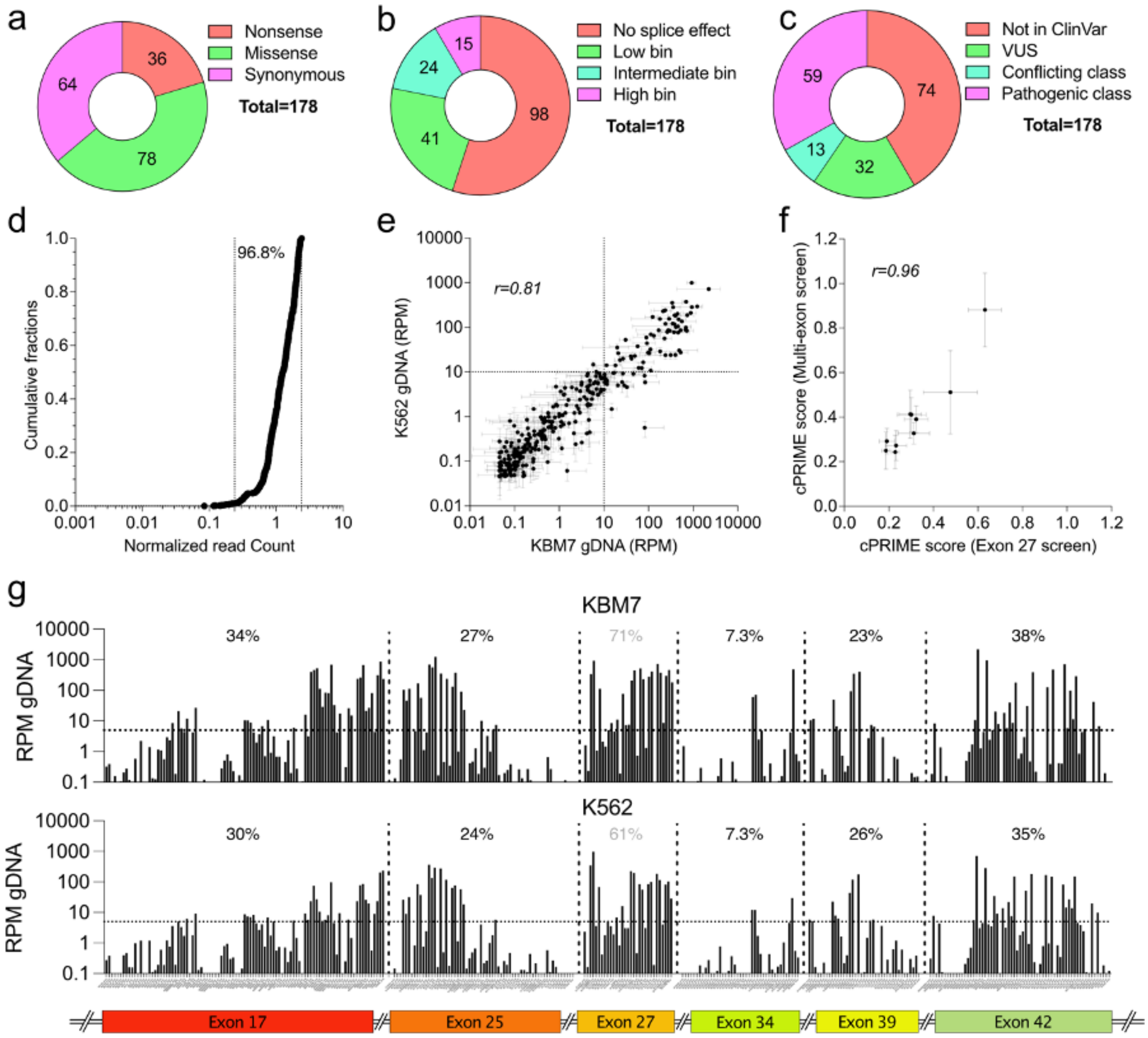

Supplementary Figure 2 - Prime editing efficiency varies per exon. a, Distribution of intended edits by variant consequence, including nonsense, missense and synonymous variants. **b,** Distribution of intended edits according to their predicted splice-gain effect by Pangolin, classified as no predicted splice effect, low, intermediate or high splice-gain bins. **c,** Distribution of intended edits according to ClinVar annotation, including variants of uncertain significance (VUS), variants with conflicting classifications of pathogenicity, variants classified as pathogenic/likely pathogenic, and variants without a ClinVar classification. Numbers indicate the number of individual intended edits in each category. **d,** Distribution of the *NF1* multi-exon pegRNA library used throughout this study. Axes as in Fig. 1e with the normalized read count (count divided by the mean pegRNA count) on the x-axis and cumulative fraction of pegRNAs on the y-axis. Dashed lines mark the 10-fold window containing 96.8% of pegRNAs, indicating even library representation. **e,** Correlation of editing frequencies between KBM7 and K562 cells. Values represent the mean of biological replicates; n=3. **f,** Correlation of cPRIME score between original Exon 27 screen and Exon 27 only from multi-exon screen. Values represent the mean of biological replicates; n=3. **g,** Prime editing frequencies in KBM7 (top) and K562 (bottom) cells at 45 days post-transduction of the pegRNA library. Functional exon 27 targeting pegRNAs were preselected from the previous screen. Percentages indicate variants with RPM > 5 from the total number of tested variants per exon. Values represent the mean of biological replicates; n=3. Source data are provided as a Source Data file.

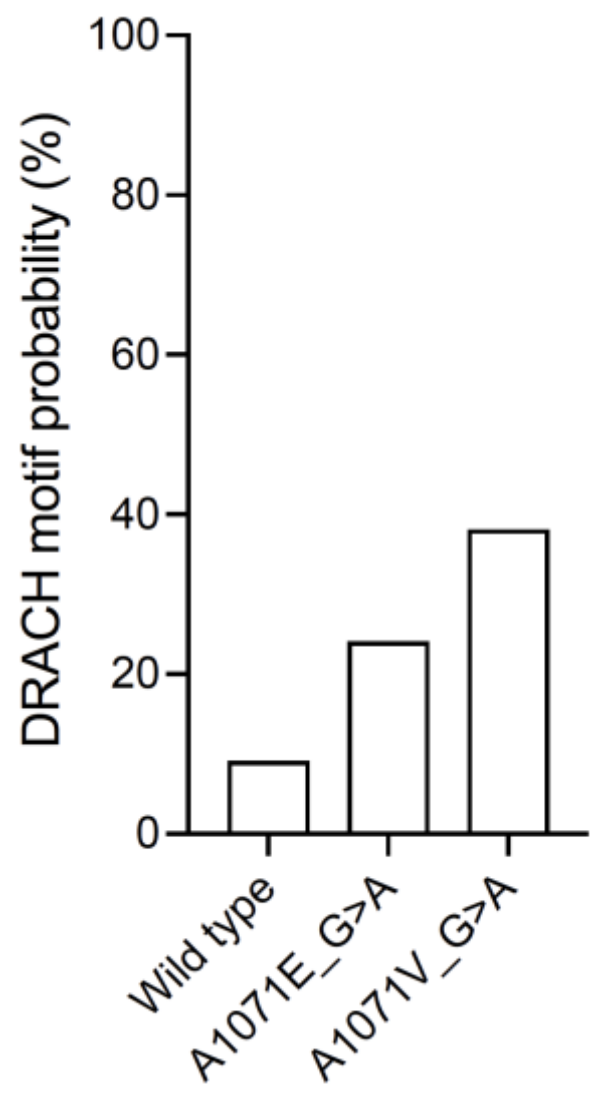

Supplementary Figure 3 - DRACH motif probability prediction using deepSRAMP for variants A1071E_G>A (edit: c.3212C>A, marker: c.3210G>A) and A1071V_G>A (edit: c.3212C>T, marker: c.3210G>A). Source data are provided as a Source Data file.

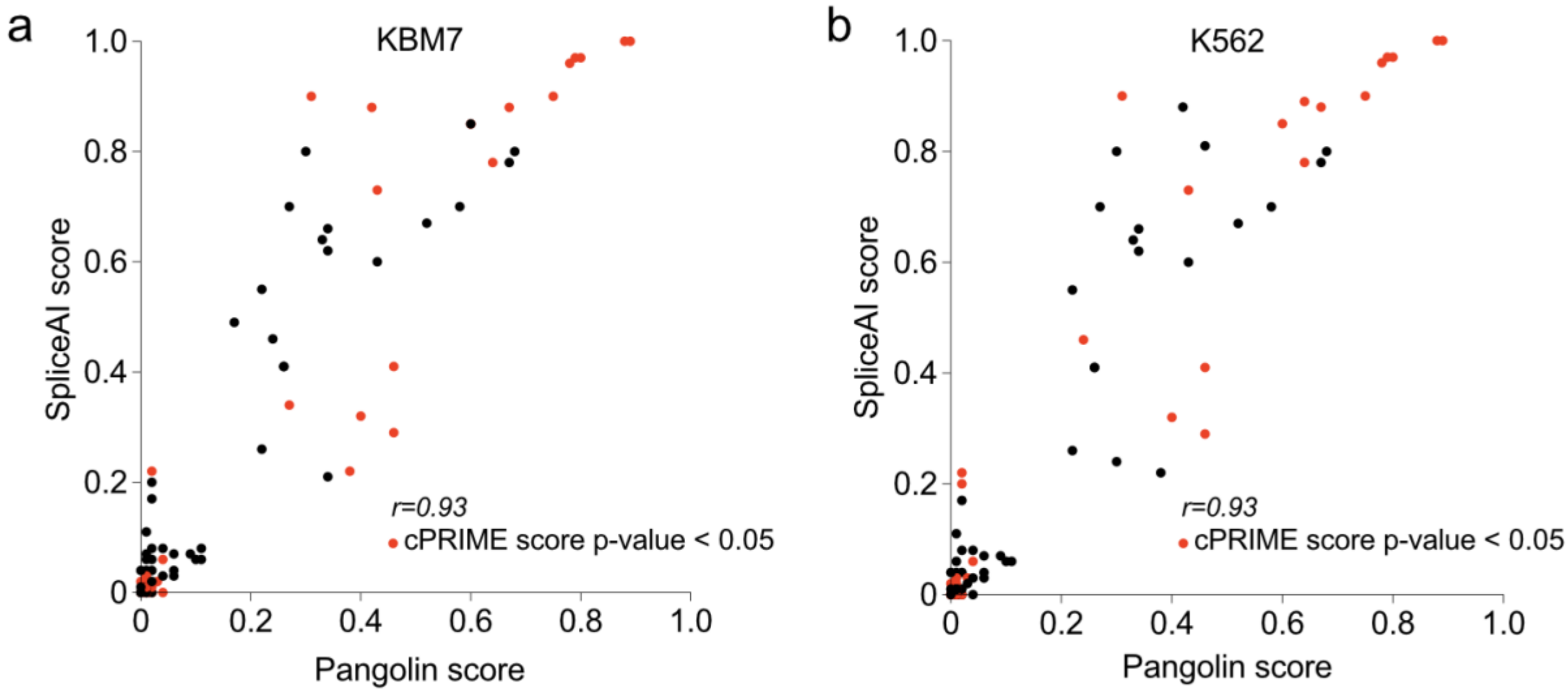

Supplementary Figure 4 - Correlation between Pangolin and SpliceAI splice-predictor algorithms. a, Correlation between Pangolin and SpliceAI splice-gain scores for all analyzed multi-exon *NF1* pegRNAs in KBM7 and **b,** K562 cells. The underlying scores are sequence-derived and identical between cell lines, so the two panels differ only in the cPRIME-hit overlay. Highlighted in red are non-STOP variants with significant ($p < 0.05$) cPRIME score <1. r values were determined by Pearson correlation. Source data are provided as a Source Data file.

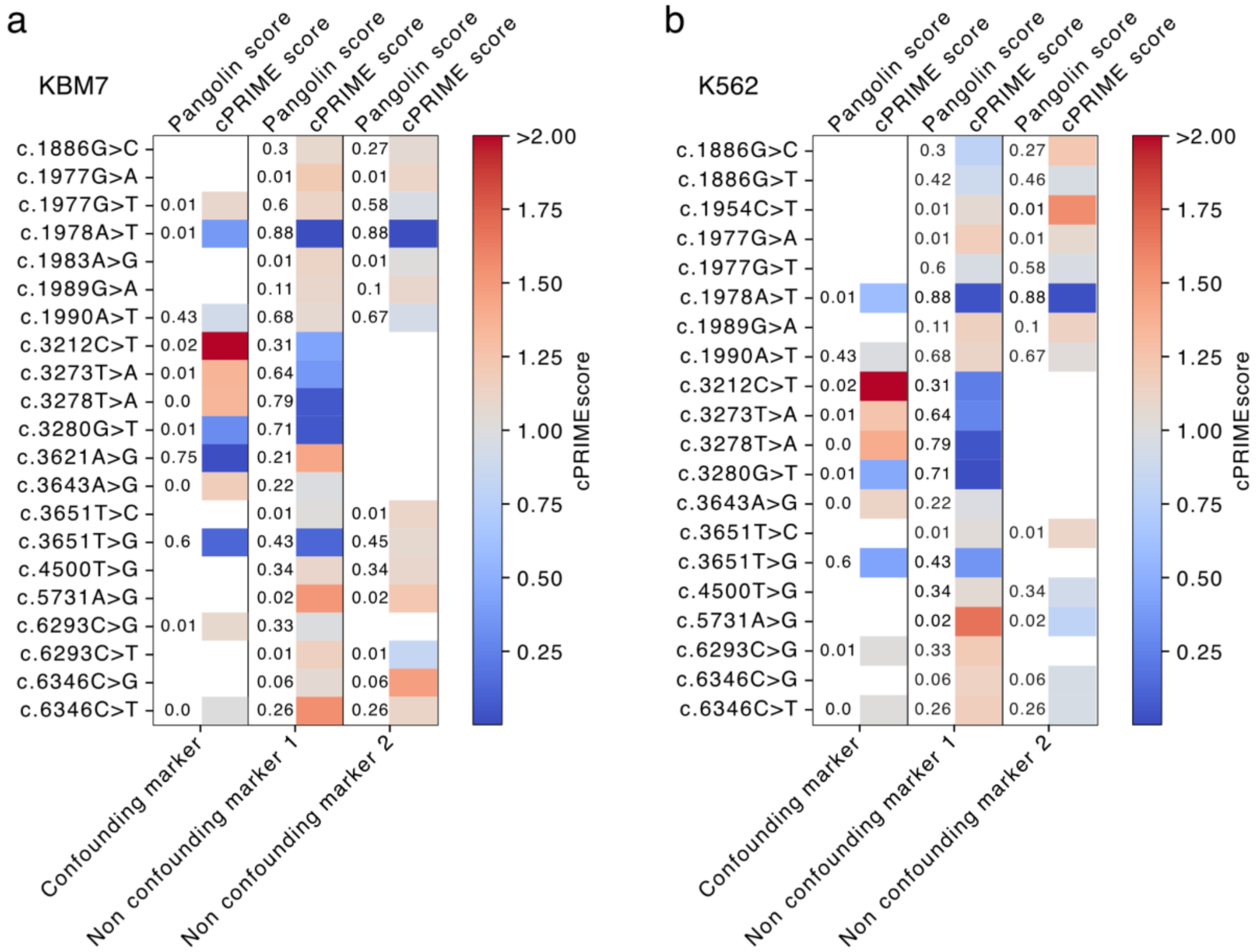

Supplementary Figure 5 - Comparison of cPRIME scores across synonymous marker edits. a, Heatmaps showing Pangolin splice-gain scores and cPRIME scores for intended edits represented by different synonymous marker edits in KBM7 and K562 **(b)** cells. For each variant, values are shown separately for pegRNAs carrying a confounding marker and 2 non-confounding markers. Only pegRNAs that passed the cutoff of RPM>5 editing efficiency on the gDNA level are shown. Pangolin scores represent the predicted splice-gain effect of the combined intended edit and synonymous marker, whereas cPRIME scores represent replicate-averaged cDNA/gDNA ratios clipped at 2 for visualization. Source data are provided as a Source Data file.

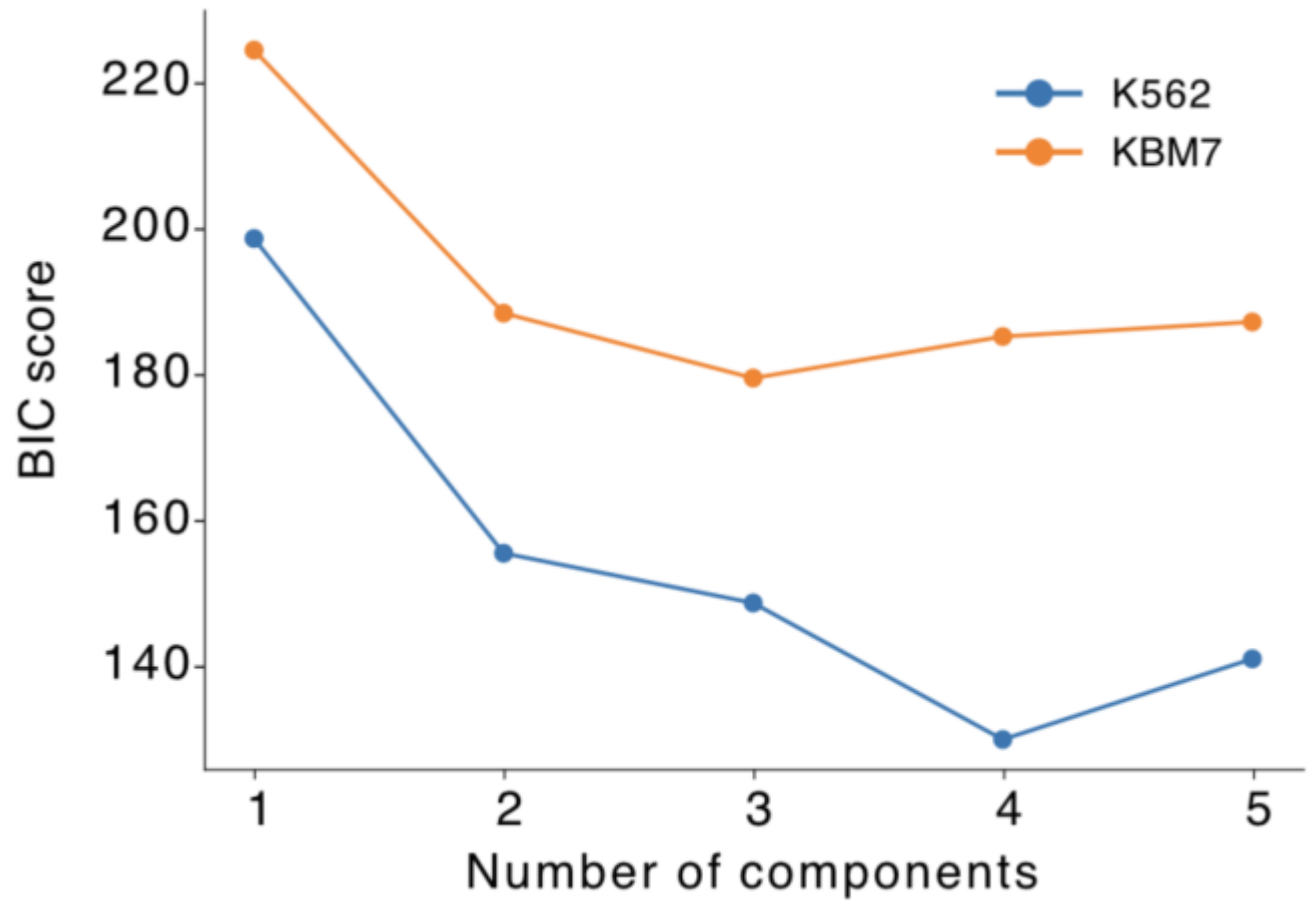

Supplementary Figure 6 - Model selection using BIC. Bayesian information criterion (BIC) scores observed for multiple numbers of GMM components fitted against cPRIME scores from K562 and KBM7 cell lines. Source data are provided as a Source Data file.
