# Supplementary material for: cPRIME enables endogenous mapping of genetic variant effects at RNA level
Source: Nat Commun. 2026 Oct 15;17:10872. doi: 10.1038/s41467-026-78344-9 (PMC13675207; doi:10.1038/s41467-026-78344-9)
Supplement: Supplementary file 2 [file 41467_2026_78344_MOESM2_ESM.pdf]

Description of Additional Supplementary Files

File Name: Supplementary Data 1
Description: Counts/RPM of gDNA and cDNA of unedited and edited KBM7 cells with or without synonymous marker edits.

File Name: Supplementary Data 2
Description: Counts/RPM of gDNA and cDNA of unedited and edited K562 cells with or without synonymous marker edits.

File Name: Supplementary Data 3
Description: Table for calculating odds ratios for KBM7 and K562 cells to check signal-to-background ratios from prime editor screens with and without synonymous marker edits.

File Name: Supplementary Data 4
Description: Counts for the PE2 Exon 27 library distribution.

File Name: Supplementary Data 5
Description: Pangolin scores for pegRNAs involved in Exon 27 screen and position R1207.

File Name: Supplementary Data 6
Description: Counts/RPM of validation experiments in KBM7 and K562 cells with and without synonymous marker edits.

File Name: Supplementary Data 7
Description: Overview table of all edits.

File Name: Supplementary Data 8
Description: Multi-exon NF1 pegRNA library, counts/RPM of gDNA and cDNA, calculated cPRIME score of each edit in K562 and KMB7 cell lines and sequences of primers used.

File Name: Supplementary Data 9
Description: GMM classification of each pegRNA from the multi-exon NF1 screen.
