# Supplementary material for: cPRIME enables endogenous mapping of genetic variant effects at RNA level
Source: Nat Commun. 2026 Oct 15;17:10872. doi: 10.1038/s41467-026-78344-9 (PMC13675207; doi:10.1038/s41467-026-78344-9)
Supplement: Supplementary file 4 [file 41467_2026_78344_MOESM5_ESM.pdf]

nature portfolio

Peer Review File

cPRIME enables endogenous mapping of genetic variant effects at RNA level

Corresponding Author: Professor Michael Boettcher

This file contains all reviewer reports in order by version, followed by all author rebuttals in order by version.

Version 0:

Reviewer comments:

Reviewer #1

(Remarks to the Author)
Summary:

The paper describes a method called cPRIME which involves using a library of pegRNAs to install variants followed by sequencing of both cDNA and gDNA to quantify each variant's effect on transcript abundance. Figure 1 establishes that marker edits are required to accurately quantify SNVs introduced at low frequencies. Figure 2 shows the results of screening nearly 100 variants in a single exon of NF1 across two cell lines. Three missense variants impacting splicing are notable hits, and effects of nonsense mediated decay (NMD) are consistently observed for nonsense variants (as expected). Figure 3 establishes that these effects validate individually, but only when introduced with certain marker edits, including the ones used in the screen.

Overall, the experimental work was carried out to a high standard. The measurements appear highly reproducible across replicates and cell lines, as well as over a broad range of pegRNA editing efficiencies. However, I was left wanting more data, as this is billed as an impactful high-throughput method. Without demonstrating an improvement to the scalability of existent methods or identifying biologically interesting variants, I found the claims of the methods utility to go too far.

Main comments:

1. The new method, cPRIME, isn't properly distinguished from existing technologies.

The novelty of the approach is that prime editing is used to deliver variants instead of HDR. Methods such as SGE (referenced by the authors) leverage the same basic cDNA-to-gDNA calculation to examine impacts of variants on RNA levels. The authors correctly point out that HDR can be limiting in many cell types, however they have not chosen cell lines in which HDR doesn't work to demonstrate their method. So what's unique here, is simply the use of PE to deliver variants. However, PE is now well-established for pooled variant delivery in studies using other readouts.

One potential advantage of cPRIME, as brought up in Discussion, is that more regions could, in principle, be tested more rapidly. However, as amplicon-seq is required to read out effects, it's unclear if this constitutes a real advantage. The use of Illumina-based amplicon-seq may preclude scoring of variants delivered to genomic regions larger than the length of Illumina sequencing reads. In this context, the potential of the method seems overstated given the authors have only analysed 98 variants. Suspect phrasings include: "potential to transform the study of RNA regulation" and a "powerful tool for the discovery of novel therapeutic targets".

2. The three missense variants scored as hits would be interesting biologically, however all do not show effects independent of the marker edits they were introduced with. While the impact of marker edits is a worthwhile point to make, as recent PE-based variant screens have featured marker edits (PMIDs: 39533107, 40580951), it leaves the paper without a novel discovery of functionally impactful human SNVs to demonstrate the method's utility.

3. It is unclear what the overall correlation with splicing predictors looks like. I would recommend showing this as it may suggest whether cPRIME or the splice-predictors are missing variants impacting splicing. For instance, all tested variants could be scored with Pangolin (both with and without) marker edits, and Pangolin scores plotted against cPRIME scores.

4. Regarding the 3 missense hits, it is particularly important to know whether splice predictors could be used to preemptively identify problematic marker edits. In line with the previous comment, Pangolin predictions for variants with each of the different marker edits shown in Figure 3 should be displayed. Furthermore, extending this analysis to additional variants (for instance, to those assayed in recently published PE screens that feature marker edits) may be a way to broaden the scope of this work.

Minor comments:

1. The following statement is confusing: “when using marker edits, intended SNPs were 1280x (KBM7) and 1120x (K562) less frequent, enabling nearly 90% of edits to be detected above background versus only 10% without marker (Fig. 1c, 1d, Supp. Table 1, 2, 3).” Please clarify whether this refers to the rate at which intended edits with marker edits are observed due to sequencing error in unedited cells (as Fig1c is labelled ‘unedited’ control)? In general, less attention is needed to the idea that multi-nucleotide variants are easier to distinguish from single-nucleotide variants, as this is well-established.

2. The reported effect of Ts on pegRNA editing efficiency is unclear. Is this specifically for 5+ consecutive Ts? If so, this should be stated clearly in Results. If not, the terminator sequence explanation is not expected to fully explain the results.

3. Missense variants impacting splicing should be written using cDNA notation, as nucleotide identity matters more than amino acid identity for splicing alterations.

4. “strand switching at poly-T sites” is not clear in meaning.

(Remarks on code availability)
I reviewed the supplementary data which report on observed variant frequencies, and the data looked realistic. I did not review any code or perform confirmatory analysis.

Reviewer #2

(Remarks to the Author)
This manuscript introduces cPRIME, a pooled prime editing-based screening framework designed to systematically evaluate the effects of single-nucleotide variants (SNVs) on RNA metabolism. The approach introduces variants at their endogenous genomic loci and quantitatively compares editing frequencies in genomic DNA (gDNA) versus complementary DNA (cDNA), thereby enabling scalable assessment of variant-induced changes in RNA stability.
In contrast to existing methodologies such as Massively Parallel Reporter Assays (MPRA) or Saturation Genome Editing (SGE), cPRIME combines the advantages of retaining endogenous genomic context with broader scalability and flexibility. Importantly, this framework enables the direct quantification of phenotypes that are not inherently linked to cell viability in pooled prime editing screens, such as RNA abundance, thereby expanding the measurable spectrum of variant effects beyond growth-based readouts. Relative to a recently reported strategy based on RNA FISH intensity and FACS binning to infer expression effects (PMID: 40245860), cPRIME directly determines variant impact through cDNA sequencing. This provides a more direct molecular readout and, in principle, resembles the sequencing-based quantification strategy used in SGE studies (PMID: 30209399).
To improve the sensitivity of pooled prime editing screens, the authors adopted synonymous marker edits, a strategy previously described (PMID: 39533107), and demonstrated their utility for distinguishing true edits from sequencing noise. Using exon 27 of the NF1 gene as a test case, they showed that cPRIME reliably detects nonsense variants subject to nonsense-mediated decay and uncovers cryptic splice-altering variants. Notably, the study reveals that although synonymous marker edits improve signal-to-background discrimination, in certain contexts these edits can themselves perturb RNA processing, representing a previously underappreciated source of confounding and an important consideration for future library design.
Taken together, these findings suggest that cPRIME holds considerable potential for both clinical and research applications. Nevertheless, in its current form, the manuscript primarily serves as a technical demonstration of the method, and it remains uncertain whether cPRIME can reliably classify the functional consequences of variants of uncertain significance (VUS) in practice. In particular, a direct comparison with existing pathogenicity data for clinically annotated variants is lacking, which would be essential to demonstrate the method’s accuracy and translational utility.
Accordingly, to strengthen the overall impact of the study, we recommend that the authors consider addressing the following points.
Major points
1. The authors limit their study to exon 27 of NF1 as the experimental model. However, no clear rationale is provided for why this particular exon was selected. If additional genomic regions are not to be tested, the authors should justify whether exon 27 is especially representative or clinically relevant. For example, is this exon known to harbor a high density of VUS, or does it play a disproportionately important role in NF1 pathogenicity? Without such justification, it is difficult to assess whether the experimental design provides sufficient evidence to support the broad applicability claimed for cPRIME. If there is no compelling justification for restricting the study to exon 27 of NF1, I would recommend including additional exons,

particularly from the well-studied RAS-GAP domain, to strengthen the manuscript's claim of general applicability. Testing a small set of additional exons would broaden the scope of the study, demonstrate that cPRIME is not limited to a single locus, and thereby increase confidence in its translational utility.

2. Through pooled screening of NF1 exon 27, the authors identify nonsense variants as well as three non-STOP variants (E1206E, R1207R, D1217E) that clearly reduce RNA abundance. However, inspection of Fig. 2d,e suggests that other variants may also influence RNA metabolism to a lesser degree, even if they fall short of being designated as "hits." At present, it is unclear how the authors determine the cutoff between true functional variants and background variation. In previous studies (e.g., Findlay et al., Nature, 2018, PMID: 30209399), quantitative frameworks and explicit thresholds were established to stratify variants into functional classes. I strongly recommend that the authors adopt a similar strategy—by defining quantitative thresholds or classification criteria to distinguish between high-confidence hits and variants with marginal effects. Providing such a framework will enhance the interpretability of the data, help avoid arbitrary classification, and increase the translational value of the method for systematic VUS annotation.

3. To establish the clinical relevance of the findings, I recommend that the authors analyze the relationship between their functional results and existing clinical pathogenicity annotations for NF1 variants as curated in databases such as ClinVar and DECIPHER. For instance, it would be highly informative to determine whether the newly identified hits (E1206E, R1207R, D1217E) have previously been reported as pathogenic or VUS in these resources. If no clinical annotation exists for these variants, I suggest expanding the study to include a broader set of NF1 variants with known clinical pathogenicity status, performing cPRIME screening for these variants, and quantitatively assessing concordance between experimental and clinical classification. Presenting metrics such as ROC curves would allow the authors to demonstrate the translational applicability and predictive performance of their approach for variant interpretation in a clinical context.

4. The authors state in Figure 3 that validation experiments were performed using cells edited with each mutation. However, simply relying on prime editing in a bulk population context is insufficient, as the editing efficiency of each pegRNA can vary substantially. Therefore, this approach may not provide adequate validation. More rigorously, it would be necessary to generate clonal cell lines harboring each individual variant and directly assess changes in RNA abundance—using methods such as qPCR or RNA-seq—rather than inferring effects from the cDNA/gDNA ratio used in the screening workflow.

Minor points

1. The term "SNP" refers specifically to variants with a population frequency greater than 1%. Since the current study deals with individual nucleotide variants regardless of their population frequency, it would be more accurate to uniformly use the term "SNV" (single nucleotide variant) rather than "SNP" throughout the manuscript.

2. In Fig. 1h–j, the authors explore determinants of prime editing efficiency and comment on variables such as poly-T content and spacer sequence identity. However, there is already substantial literature systematically addressing the sequence features that impact prime editing efficiency—for example, GC content of the PBS or computationally predicted efficiency scores for the 20nt spacer (see, e.g., Yu et al., Cell, 2023, PMID: 37119812; Mathis et al., Nat Biotechnol, 2024, PMID: 38907037). Several algorithms have been developed to predict editing efficiency based on these parameters. To enhance the rigor and generalizability of these findings, I would recommend that the authors quantitatively analyze the correlation between their observed editing frequencies and the scores predicted by these established algorithms. Such analysis would provide more meaningful context for the observed variability in editing efficiency and more robustly inform future pegRNA library design strategies.

3. The authors state in Figures 3e and 3f that a reduction in RNA abundance was observed only when the intended edit was combined with the synonymous marker in the R1207R variant. Has the resulting sequence in these cases been reported as a motif known to induce changes in RNA splicing?

4. On page 5, the authors claim: "Such an approach would eliminate the need for separate libraries, as required by SGE, and hence allow the simultaneous investigation of both exonic regions, as demonstrated in this study, and non-coding expressed regions such as untranslated regions (UTRs) or long non-coding RNAs." However, cPRIME relies on simultaneous sequencing of target genomic DNA and cDNA, typically using Illumina platforms, which can generally capture sequences up to 300bp in length. If the targeted genomic region or the cDNA itself is substantially longer than this limit, the approach may still be constrained to analyzing short amplicons, and additional library preparations would be necessary for comprehensive coverage of longer regions. Supporting this concern, previous literature (e.g., PMID: 39533107) has shown that even for prime editing-based screens, separate libraries are often generated for individual exons, particularly when targeting large or multi-exon genes. Therefore, the claim that cPRIME universally eliminates the need for separate libraries is not fully convincing in practice, especially when analyzing long transcripts or large genomic regions. Given the method's reliance on high sequencing accuracy and short-read platforms, it is unclear that separate libraries can be universally avoided as the authors suggest.

5. In the Methods section: "KBM7_PE_MLH1 cells were transduced with lentivirally packaged exon 27 library at multiplicity of infection (MOI) =0.7 and 100.000x cell coverage as triplicates..." In this context, do "100.000x" and "10.000x" refer to "100,000×" and "10,000×" respectively? The notation should be clarified for consistency with standard scientific convention.

(Remarks on code availability)

Reviewer #3

(Remarks to the Author)

Westphal et al. present a study using a small prime editing library to introduce variants in exon 27 of NF1. By comparing variant read counts from gDNA and cDNA, the authors measured both prime editing efficiency and the transcript-level consequences of editing. They validated that introducing nonsense or splice-disrupting variants reduces transcript abundance. The manuscript is clearly written, and the figures and data presentation are straightforward to follow. The

attempt to incorporate transcript-level readouts in prime editing screening is interesting, and the discussion on the potential confounding effects of synonymous marker edits is a valuable contribution to the field. Nevertheless, while these aspects are useful, the overall cPRIME platform is constrained by low efficiency, small scale, and unclear advantages over existing methods.

Major comments:
1. The current work is restricted to ~100 pegRNAs targeting a single exon, with extremely low editing efficiencies. The methods are clearly described and appear reproducible, but in terms of target scope, time, and cost of screening, the study does not offer clear advantages over existing prime editing platforms (PMID: 38134886, 38472508, 39533107, 40580951). It would be helpful if the manuscript explicitly discussed potential distinctions and advantages compared to these existing platforms.

2. Screening ~50 variants in a single exon is insufficient to support claims of a broadly applicable platform. At minimum, results from an additional exon or gene, or a concrete strategy for scaling to larger variant sets, would be necessary.

3. The use of cDNA readouts to analyze RNA metabolism (e.g., NMD, splicing) is conceptually novel in prime editing screening. However, the contribution remains limited, as prior MPRA-based approaches (PMID: 35051175, 37192163, 40715118) have already enabled high-throughput splicing variant analysis. To my knowledge, chromatin status has little impact on splicing outcomes, making it unclear whether cPRIME provides meaningful advantages over MPRA. In fact, cPRIME requires the generation of PE-expressing, MMR-deficient cell lines, which may make the system more complex rather than more practical. A direct performance comparison with MPRA would strengthen the manuscript.

(Remarks on code availability)

Version 1:

Reviewer comments:

Reviewer #1

(Remarks to the Author)
The authors have done well to revise their manuscript. Most importantly, they've added a considerable amount of data in a new experiment, showing that their approach can be applied to multiple exons at the same time, which constitutes a unique advantage of cPRIME. Thanks for taking the reviewer feedback onboard and improving the manuscript.

The addition of new data is much appreciated, but the major limitation of the manuscript is still the relatively low throughput of the experiments compared to other methods. The maximum library size remains under 200 variants / 400 pegRNAs. Therefore, while the technical quality of the data looks alright, it's still not established to what extent cPRIME may be scaled up further. Despite this major limitation, I find the method's strengths and weaknesses to be well characterised within the context of the current literature. Therefore, with some additional revisions I would recommend this for publication.

1. Regarding "Within the cPRIME workflow, such transcripts may be underrepresented during cDNA amplification and amplicon purification, for example due to loss of primer binding sites or the generation of shorter splice isoforms.":

Please specify here exactly how the cPRIME score is calculated. Specifically, if a variant is observed in cDNA on an alternative splice form (arising due to altered splicing), do those reads contribute to the variant's cPRIME score, or are only reads used if they map to the canonically spliced transcript? I ask because in many use cases it will be important to know whether low cPRIME scores reliably mean loss-of-function due to low transcript levels, or whether alternative "rescue" transcripts may arise that will not be accounted for in the score.

2. Sample sizes are required throughout Figure 4 to place the performance metrics in context. For instance, it appears as if the ROC-AUC analysis was performed using very few gold-standard variants, based on the following panels. This is essential information, so please add n = x for each group to the figures themselves.

3. Related to the point above, I was surprised to see the method work so well for ClinVar Pathogenic variants because many pathogenic variants will not impact splicing (for instance, most missense variants). Reading the methods, it became clear that variants were pre-selected for testing due to potentially having an impact on RNA levels. Therefore, please make it explicit in the Results which ClinVar variants were included in testing, and what the criteria was applied to prioritise them.

4. The authors attempt to classify variants into one of three functional categories using the GMM. What was the rationale for having 3 categories instead of 2? It seems most critical to establish the score threshold below which variants are highly likely to be pathogenic, in line with an ACMG-style calibration (Brnich et al 2019). Currently, the "responder" category seems to include only variants with a cPRIME score < -2 (log10 scale), corresponding to a 99% depletion in RNA. Is this correct? This seems much lower than what may be necessary for identifying pathogenic, splice-disruptive variants. Meanwhile the intermediate category is quite broad and includes far more variants (20/15 vs 7). Not sure whether this was the case here, but as nonsense variants impact function via premature protein truncation, these should be excluded from any analysis that attempts to calibrate the scores to clinical pathogenicity.

5. I found the contrast between black and gray difficult to appreciate in Fig3f. Further, it would be good to use a scheme that also discriminates which variants were hits (as in d, e), because ultimately the reader is keen to know how this impacts hits.

6. I would certainly change the “responder” vs. “non-responder” terminology to something more consistent with established ways of describing variant effects. For instance, “RNA-depleted”, “RNA-intermediate”, “RNA-neutral”, maybe? (To me, ‘Responder’ sounds like it relates to a drug response.)

(Remarks on code availability)

Reviewer #2

(Remarks to the Author)
Overall, the revised manuscript has been substantially improved, and I appreciate the authors’ efforts to address the previous comments. In particular, the additional multi-exon analysis, the clearer discussion of marker-dependent effects, and the introduction of a quantitative classification framework have strengthened the study. I do not think additional experiments are necessary at this stage, but I have a few suggestions that may further improve the clarity and completeness of the manuscript.

1. Regarding the statements, “By eliminating the need for locus-specific DNA donor template pools required for SGE, cPRIME enables variants across multiple genomic loci to be installed from a single pegRNA library” and “Thus, cPRIME enables pooled installation and screening of variants across multiple genomic loci using a single pegRNA library, while the sequencing readout remains target-specific and must be adapted to the genomic and transcript architecture of the region under investigation.”
These revisions are helpful and provide a more balanced description of the current scope of the method. At the same time, it would be useful to discuss more explicitly the trade-off between breadth and effective per-target coverage in the multi-exon format. As shown in Fig. 3c, the fraction of variants detected above threshold remains relatively modest and uneven across exons, ranging from 7.3% to 38% in KBM7 cells and from 7.3% to 34% in K562 cells. This suggests that, as library complexity increases, effective coverage for individual target regions may become limiting, particularly given the low and variable prime-editing efficiencies observed here. In such settings, region-specific or exon-focused libraries may be preferable in some cases to help preserve coverage and sensitivity. A somewhat fuller discussion of this trade-off would strengthen the manuscript.

2. The Pangolin-based analyses are a useful part of the manuscript and help interpret many of the cPRIME score hits. Nevertheless, because the study relies so heavily on a single splice predictor, it would further strengthen the manuscript if the authors could show that similar conclusions are supported by at least one independent predictor, such as SpliceAI (PMID: 30661751). This would help demonstrate that the main interpretations are robust to predictor choice rather than being specific to Pangolin.

3. Regarding the statement, “The library showed a narrow distribution, with 96.8% of pegRNAs falling within one order of magnitude (Supp. Fig. 2d).”
The intended meaning of this sentence remains somewhat unclear. It would be helpful if the authors could clarify in the Results text what the normalized read counts refer to, what sample or library was sequenced to obtain them, and how the “within one order of magnitude” criterion was defined. Making the corresponding figure legend more explicit would also improve readability.

4. The terminology should be revised consistently throughout the manuscript. “SNP” still appears in Fig. 1c and the Methods, despite the previous request to replace this term with “SNV.”

5. Regarding the statement, “Interestingly, several variants, most notably, A1071E_G>A (edit: c.3212C>A, marker: c.3210G>A) and A1071V_G>A (edit: c.3212C>T, marker: c.3210G>A) showed significantly increased cPRIME scores in both cell lines, suggesting increased relative abundance of the edited transcript (Fig. 3c). Additional experiments would be required to illuminate the underlying mechanism.”
These observations are intriguing. While additional mechanistic experiments are likely beyond the scope of the present study, it would strengthen the manuscript if the authors could provide a somewhat fuller discussion of plausible explanations, such as altered RNA processing, increased transcript stability, or possible marker-related effects, and indicate whether any in silico analyses support these hypothesis.

6. The Gaussian mixture model-based classification is a useful addition. For reproducibility, the Methods should specify the covariance structure used for the GMM, for example whether the components were modeled with shared or component-specific covariance.

(Remarks on code availability)

Reviewer #3

(Remarks to the Author)

In the revised manuscript, the authors have thoroughly addressed my concerns through additional wet-lab experiments and computational analyses. In particular, the multi-exon screen and the comparison with ClinVar pathogenicity annotations substantially strengthen the claim of general applicability, and the repositioning of cPRIME relative to existing platforms is now much clearer. This work would be highly beneficial for a wide range of readers in the functional genomics and variant interpretation field.

(Remarks on code availability)

Version 2:

Reviewer comments:

Reviewer #1

(Remarks to the Author)
Thanks to the authors for submitting a further improved revision of their manuscript. My previous concerns have been sufficiently addressed. I particularly appreciate the more clear explanation of the cPRIME score derivation and the inclusion of sample sizes in the relevant Figure panels. I do not think that any further revisions are required.

(Remarks on code availability)

Reviewer #2

(Remarks to the Author)
Overall, the authors have addressed my previous comments satisfactorily, and the revised manuscript is substantially improved. In particular, the additional multi-exon analysis, the improved discussion of marker-related confounding, the added comparison with an independent splice predictor, and the clarification of the quantitative analysis framework all contribute to a stronger and more complete manuscript. I do not think any further revisions are necessary at this stage.

(Remarks on code availability)

Open Access This Peer Review File is licensed under a Creative Commons Attribution 4.0 International License, which permits use, sharing, adaptation, distribution and reproduction in any medium or format, as long as you give appropriate credit to the original author(s) and the source, provide a link to the Creative Commons license, and indicate if changes were made.
In cases where reviewers are anonymous, credit should be given to 'Anonymous Referee' and the source.
The images or other third party material in this Peer Review File are included in the article’s Creative Commons license, unless indicated otherwise in a credit line to the material. If material is not included in the article’s Creative Commons license and your intended use is not permitted by statutory regulation or exceeds the permitted use, you will need to obtain

permission directly from the copyright holder.
To view a copy of this license, visit https://creativecommons.org/licenses/by/4.0/

cPRIME enables endogenous mapping of genetic variant effects at RNA level

Ghanem El Kassem[1*], Constanze Westphal[1*], Marvin Doebel[1*], Jasmine Hillmer[1], Tanja Seifert[1], Michael Boettcher[1#]

[1] Institute of Molecular Medicine, Section for Molecular Medicine of Signal Transduction, Faculty of Medicine, Martin Luther University Halle-Wittenberg, 06120 Halle, Germany

[#]Correspondence to: michael.boettcher@medizin.uni-halle.de

*equal contribution

We would like to thank the reviewers for their careful evaluation of our manuscript and for their constructive comments, which helped us substantially improve the scope, clarity, and interpretation of the study. Below, we respond point by point to the reviewers' comments.

REVIEWER COMMENTS

Reviewer #1 (Remarks to the Author):

Summary:

The paper describes a method called cPRIME which involves using a library of pegRNAs to install variants followed by sequencing of both cDNA and gDNA to quantify each variant's effect on transcript abundance. Figure 1 establishes that marker edits are required to accurately quantify SNVs introduced at low frequencies. Figure 2 shows the results of screening nearly 100 variants in a single exon of NF1 across two cell lines. Three missense variants impacting splicing are notable hits, and effects of nonsense mediated decay (NMD) are consistently observed for nonsense variants (as expected). Figure 3 establishes that these effects validate individually, but only when introduced with certain marker edits, including the ones used in the screen.

Overall, the experimental work was carried out to a high standard. The measurements appear highly reproducible across replicates and cell lines, as well as over a broad range of pegRNA editing efficiencies. However, I was left wanting more data, as this is billed as an impactful high-throughput method. Without demonstrating an improvement to the scalability of existent methods or identifying biologically interesting variants, I found the claims of the methods utility to go too far.

Main comments:

1. The new method, cPRIME, isn't properly distinguished from existing technologies.

The novelty of the approach is that prime editing is used to deliver variants instead of HDR. Methods such as SGE (referenced by the authors) leverage the same basic cDNA-to-gDNA calculation to examine impacts of variants on RNA levels. The authors correctly point out that HDR can be limiting in many cell types, however they have not chosen cell lines in which HDR

doesn't work to demonstrate their method. So what's unique here, is simply the use of PE to deliver variants. However, PE is now well-established for pooled variant delivery in studies using other readouts.

One potential advantage of cPRIME, as brought up in Discussion, is that more regions could, in principle, be tested more rapidly. However, as amplicon-seq is required to read out effects, it's unclear if this constitutes a real advantage. The use of Illumina-based amplicon-seq may preclude scoring of variants delivered to genomic regions larger than the length of Illumina sequencing reads. In this context, the potential of the method seems overstated given the authors have only analysed 98 variants. Suspect phrasings include: "potential to transform the study of RNA regulation" and a "powerful tool for the discovery of novel therapeutic targets".

We thank the reviewer for this important comment. We agree that cPRIME should have been more clearly positioned relative to existing technologies, particularly HDR-based saturation genome editing approaches that also use cDNA-to-gDNA comparisons to quantify effects on RNA abundance. We have therefore revised the Introduction and Discussion to clarify that cPRIME builds on this established principle, rather than representing a conceptually new readout strategy. In the revised manuscript, we now emphasize that the main distinction of cPRIME is the use of prime editing to install variants in pooled format without donor templates or double-strand breaks.

We have also toned down broader statements regarding the future impact of cPRIME. In particular, we removed stronger phrasing such as "*transform the study of RNA metabolism*" and "*powerful tool for the discovery of novel therapeutic targets*" and replaced it with more measured language.

We also addressed this Reviewer concern experimentally by performing an additional cPRIME screen targeting multiple NF1 exons (Fig. 3a–c). This demonstrated that cPRIME can be applied to multiple distinct genomic loci using a single pegRNA library. However, amplicon sequencing remains an intrinsic part of cPRIME, because the assay is designed to measure variant effects on RNA abundance and processing. Unlike proliferation-based variant screens, which rely on altered protein function being coupled to cell fitness, RNA-level effects measured by cPRIME cannot generally be assessed through a proliferation readout. We now also explicitly discuss the constraints imposed by amplicon sequencing in the revised manuscript.

Together, these revisions more clearly define the relationship between cPRIME and existing approaches, while providing a more balanced description of the method's future potential.

2. The three missense variants scored as hits would be interesting biologically, however all do not show effects independent of the marker edits they were introduced with. While the impact of marker edits is a worthwhile point to make, as recent PE-based variant screens have featured marker edits (PMIDs: 39533107, 40580951), it leaves the paper without a novel discovery of functionally impactful human SNVs to demonstrate the method's utility.

We acknowledge that the dependence of the three initially identified non-STOP hits on their associated marker edits limits their biological interpretation as independent variant effects. We have therefore revised the manuscript to make this point explicit and no longer present these variants as biologically interpretable hits independent of the marker context. Instead, we now use them to demonstrate an important technical consideration for prime-editing screens: synonymous marker edits can themselves alter RNA processing and therefore need to be carefully evaluated during library design.

To address the broader concern that the manuscript lacks functionally informative human variants, we performed an additional cPRIME screen targeting five further NF1 exons, thereby expanding the analysis beyond the original exon 27 screen (Fig. 3a–c). For this expanded screen, we incorporated Pangolin-based prefiltering to identify pegRNA designs in which the synonymous marker edit was predicted to confound the splice effect of the intended variant (Fig. 3f). Marker-sensitive designs were excluded from downstream variant classification, allowing us to focus on variants whose cPRIME scores are less likely to be driven by marker-induced splice effects. This expanded analysis identified additional RNA-level variant effects across multiple NF1 exons and enabled comparison with ClinVar

pathogenicity annotations (Fig. 4), thereby providing a stronger demonstration of cPRIME's utility for interpreting RNA-altering variants.

We also agree that marker edits have been used in recent prime-editing-based variant screens, and we cited these studies in the original and current submission. We do not claim that marker edits are unique to cPRIME; rather, our data highlight both their practical value and their potential risk. In cPRIME, marker edits currently improve sensitivity because individual prime-edited SNVs can occur at frequencies too low to quantify reliably without a linked, more readily distinguishable marker edit (Fig. 1b–d). At the same time, our data show that marker edits must be computationally assessed and, where necessary, experimentally controlled. We have therefore clarified that biological interpretation of individual variant hits requires marker-aware filtering, independent marker designs, and/or validation without the marker edit.

3. It is unclear what the overall correlation with splicing predictors looks like. I would recommend showing this as it may suggest whether cPRIME or the splice-predictors are missing variants impacting splicing. For instance, all tested variants could be scored with Pangolin (both with and without) marker edits, and Pangolin scores plotted against cPRIME scores.

In the revised manuscript, we now directly compare Pangolin splice-gain scores with cPRIME scores. For the original NF1 exon 27 screen, this analysis is shown in Fig. 2g,h for KBM7 and K562 cells, respectively. For the expanded NF1 multi-exon screen, the corresponding analysis is shown in Fig. 3d,e. These analyses show that variants with high Pangolin scores are enriched among variants with reduced cPRIME scores, consistent with splice-associated transcript depletion. At the same time, several variants show discordance between experimental cPRIME measurements and computational predictions (especially in the mid-ranges of Pangolin scores), supporting the value of experimental validation.

Finally, in the expanded NF1 multi-exon screen, we used Pangolin during library design to identify marker edits predicted to confound the splice effect of the intended variant. These marker-sensitive designs were flagged and excluded from downstream analyses focused on classifying intended variant effects (Fig. 3f). Together, these analyses address the reviewer's suggestion by showing the overall relationship between cPRIME and splice-prediction scores, while also demonstrating how splice predictors can be used to improve marker-edit design and interpretation.

4. Regarding the 3 missense hits, it is particularly important to know whether splice predictors could be used to preemptively identify problematic marker edits. In line with the previous comment, Pangolin predictions for variants with each of the different marker edits shown in Figure 3 should be displayed. Furthermore, extending this analysis to additional variants (for instance, to those assayed in recently published PE screens that feature marker edits) may be a way to broaden the scope of this work.

We agree that splice-site predictors could be valuable for identifying potentially problematic marker edits before library construction. To address this point, we now compare Pangolin scores with cPRIME scores across both the original NF1 exon 27 screen and the expanded NF1 multi-exon screen, as shown in revised Fig. 2g,h and Fig. 3d,e. We also incorporated Pangolin-based prefiltering into the design and analysis of the multi-exon screen to identify pegRNA designs in which the marker edit itself was predicted to confound the effect of the intended variant. These marker-sensitive designs were flagged in Fig. 3f and excluded from downstream analyses focused on ClinVar-based variant classification. Together, these additions support the reviewer's suggestion that splice predictors can be used during pegRNA library design to identify marker edits that may introduce or modify splice motifs.

Regarding the suggestion to extend this analysis to recently published prime-editing screens using marker edits, we agree that this would be an interesting broader analysis. However, to our knowledge,

currently available prime-editing screens using marker edits do not include a matched cDNA/RNA-level readout analogous to cPRIME. As a result, they do not allow direct assessment of whether marker edits alter RNA processing or confound cDNA/gDNA-based variant-effect measurements. Instead, we addressed this point experimentally within our own system, as explained above.

Minor comments:

1. The following statement is confusing: "when using marker edits, intended SNPs were 1280x (KBM7) and 1120x (K562) less frequent, enabling nearly 90% of edits to be detected above background versus only 10% without marker (Fig. 1c, 1d, Supp. Table 1, 2, 3)." Please clarify whether this refers to the rate at which intended edits with marker edits are observed due to sequencing error in unedited cells (as Fig1c is labelled 'unedited' control)? In general, less attention is needed to the idea that multi-nucleotide variants are easier to distinguish from single-nucleotide variants, as this is well-established.

We have revised the text to clarify that the 1280-fold and 1120-fold differences refer to the edit-level background frequencies measured in unedited control cells, not to editing frequencies in prime-edited samples. As expected, the combined edit-marker haplotype showed a much lower background frequency than the edit alone, allowing marker-linked edits to be quantified above background for a larger fraction of variants. We also make it clear that the principle of synonymous marker edits has been developed and published by other groups.

2. The reported effect of Ts on pegRNA editing efficiency is unclear. Is this specifically for 5+ consecutive Ts? If so, this should be stated clearly in Results. If not, the terminator sequence explanation is not expected to fully explain the results.

We agree that the statement should specify that the observed effect refers to stretches of thymidines rather than thymidine content in general. We have revised the Results section to clarify that the reduced editing efficiency was associated with pegRNAs containing >4 consecutive thymine residues, consistent with the known function of poly-T stretches as polymerase III termination signals. This observation is also consistent with the recent report by Oh et al. 2026 (now also cited in the manuscript).

3. Missense variants impacting splicing should be written using cDNA notation, as nucleotide identity matters more than amino acid identity for splicing alterations.

We agree that cDNA notation is more informative for splice-altering variants, as their functional impact depends on the underlying nucleotide change rather than the resulting amino acid annotation. We therefore added HGVSc identifiers for relevant variants likely to affect splicing. In addition, we added the genomic and cDNA positions of both the intended edit and the synonymous marker variant to Supplementary Tables 7 and 8.

4. "strand switching at poly-T sites" is not clear in meaning.

We agree with the reviewer that the phrase "strand switching at poly-T sites" was unclear. What we intended to describe was the selection of alternative pegRNA designs, including pegRNAs targeting the opposite DNA strand, when this avoids extended thymine stretches in the expressed pegRNA sequence. We have therefore removed the ambiguous phrase "strand switching" and revised the text to describe more generally that poly-T stretches in pegRNAs should be avoided, as they may cause premature termination of U6-driven transcription and reduce editing efficiency.

Reviewer #1 (Remarks on code availability):

I reviewed the supplementary data which report on observed variant frequencies, and the data looked realistic. I did not review any code or perform confirmatory analysis.

Reviewer #2 (Remarks to the Author):

This manuscript introduces cPRIME, a pooled prime editing-based screening framework designed to systematically evaluate the effects of single-nucleotide variants (SNVs) on RNA metabolism. The approach introduces variants at their endogenous genomic loci and quantitatively compares editing frequencies in genomic DNA (gDNA) versus complementary DNA (cDNA), thereby enabling scalable assessment of variant-induced changes in RNA stability.
In contrast to existing methodologies such as Massively Parallel Reporter Assays (MPRA) or Saturation Genome Editing (SGE), cPRIME combines the advantages of retaining endogenous genomic context with broader scalability and flexibility. Importantly, this framework enables the direct quantification of phenotypes that are not inherently linked to cell viability in pooled prime editing screens, such as RNA abundance, thereby expanding the measurable spectrum of variant effects beyond growth-based readouts. Relative to a recently reported strategy based on RNA FISH intensity and FACS binning to infer expression effects (PMID: 40245860), cPRIME directly determines variant impact through cDNA sequencing. This provides a more direct molecular readout and, in principle, resembles the sequencing-based quantification strategy used in SGE studies (PMID: 30209399).
To improve the sensitivity of pooled prime editing screens, the authors adopted synonymous marker edits, a strategy previously described (PMID: 39533107), and demonstrated their utility for distinguishing true edits from sequencing noise. Using exon 27 of the NF1 gene as a test case, they showed that cPRIME reliably detects nonsense variants subject to nonsense-mediated decay and uncovers cryptic splice-altering variants. Notably, the study reveals that although synonymous marker edits improve signal-to-background discrimination, in certain contexts these edits can themselves perturb RNA processing, representing a previously underappreciated source of confounding and an important consideration for future library design.
Taken together, these findings suggest that cPRIME holds considerable potential for both clinical and research applications. Nevertheless, in its current form, the manuscript primarily serves as a technical demonstration of the method, and it remains uncertain whether cPRIME can reliably classify the functional consequences of variants of uncertain significance (VUS) in practice. In particular, a direct comparison with existing pathogenicity data for clinically annotated variants is lacking, which would be essential to demonstrate the method's accuracy and translational utility. Accordingly, to strengthen the overall impact of the study, we recommend that the authors consider addressing the following points.

Major points

1. The authors limit their study to exon 27 of NF1 as the experimental model. However, no clear rationale is provided for why this particular exon was selected. If additional genomic regions are not to be tested, the authors should justify whether exon 27 is especially representative or clinically relevant. For example, is this exon known to harbor a high density of VUS, or does it play a disproportionately important role in NF1 pathogenicity? Without such justification, it is difficult to assess whether the experimental design provides sufficient evidence to support the broad applicability claimed for cPRIME. If there is no compelling justification for restricting the study to exon 27 of NF1, I would recommend including additional exons, particularly from the well-studied RAS-GAP domain, to strengthen the manuscript's claim of general applicability. Testing a small set of additional exons would broaden the scope of the study, demonstrate that cPRIME is not limited to a single locus, and thereby increase confidence in its translational utility.

We thank the reviewer for this important point regarding the choice of exon 27 and the generalizability of our approach. As now explained in the manuscript, exon 27 of NF1 was selected as an initial model exon because it lies within the central GTPase-activating protein-related domain (GRD), a functionally important region of NF1. At the same time, exon 27 was not selected based on a known RNA-level phenotype, making it suitable as an unbiased proof-of-concept target for testing whether cPRIME can detect variant effects on RNA abundance and processing.

To address the reviewer's concern regarding general applicability, we extended our analysis beyond exon 27 by performing an additional screen targeting five further NF1 exons with high densities of clinically annotated variants and predicted splice-altering events. Across these additional regions, we observed consistent RNA-level depletion associated with predicted splice-disrupting variants and nonsense-mediated decay (Fig. 3). These findings support the applicability of cPRIME beyond a single exon.

Taken together, exon 27 served as a test case, while the expanded multi-exon screen demonstrates that cPRIME is not limited to a single genomic locus but can be applied to multiple distant genomic regions using a single pegRNA library.

2. Through pooled screening of NF1 exon 27, the authors identify nonsense variants as well as three non-STOP variants (E1206E, R1207R, D1217E) that clearly reduce RNA abundance. However, inspection of Fig. 2d,e suggests that other variants may also influence RNA metabolism to a lesser degree, even if they fall short of being designated as "hits." At present, it is unclear how the authors determine the cutoff between true functional variants and background variation. In previous studies (e.g., Findlay et al., Nature, 2018, PMID: 30209399), quantitative frameworks and explicit thresholds were established to stratify variants into functional classes. I strongly recommend that the authors adopt a similar strategy—by defining quantitative thresholds or classification criteria to distinguish between high-confidence hits and variants with marginal effects. Providing such a framework will enhance the interpretability of the data, help avoid arbitrary classification, and increase the translational value of the method for systematic VUS annotation.

We thank the reviewer for this important suggestion regarding the need for a quantitative framework to distinguish functional variants from background variation. We agree that explicit and data-driven classification criteria are essential to improve interpretability and avoid arbitrary thresholds.

In response, we implemented an unsupervised Gaussian mixture model (GMM)–based approach to classify variants based on their cPRIME scores. This framework models the empirical distribution of scores and assigns variants to responder, intermediate, and non-responder classes based on posterior probabilities, thereby defining thresholds in a data-driven manner.

The number of components was evaluated using the Bayesian Information Criterion (BIC). A three-component model was optimal in the KBM7 dataset, while in K562 a four-component model provided only a marginal improvement in BIC. Importantly, classification was highly concordant between three- and four-component models in K562 (65/67 variants assigned to the same class), with the additional component primarily capturing a single outlier rather than defining a distinct functional category. Based on these results and the expectation of three biologically meaningful classes (responder, non-responder, intermediate), we selected a three-component model for further analyses.

To ensure robust model fitting, we further applied mild clipping of extreme cPrime values to limit the influence of outliers. This preprocessing affected only a small number of variants and did not materially impact classification (94–100% concordance with unclipped data).

These additions are now described in their respective Results and Methods sections and supported by a new supplementary figure (Supp. Fig. 4). Overall, this revised framework provides a principled and reproducible data-driven strategy for variant classification, consistent with prior quantitative approaches such as Findlay et al. Nature 2018.

3. To establish the clinical relevance of the findings, I recommend that the authors analyze the relationship between their functional results and existing clinical pathogenicity annotations for NF1 variants as curated in databases such as ClinVar and DECIPHER. For instance, it would be highly informative to determine whether the newly identified hits (E1206E, R1207R, D1217E) have previously been reported as pathogenic or VUS in these resources. If no clinical annotation exists for these variants, I suggest expanding the study to include a broader set of NF1 variants

with known clinical pathogenicity status, performing cPRIME screening for these variants, and quantitatively assessing concordance between experimental and clinical classification. Presenting metrics such as ROC curves would allow the authors to demonstrate the translational applicability and predictive performance of their approach for variant interpretation in a clinical context.

We thank the reviewer for this excellent suggestion to assess the relationship between our functional measurements and existing clinical variant annotations.

In response, we performed a quantitative comparison between cPRIME scores and clinical pathogenicity classifications from ClinVar from our multi-exon screen. Variants annotated as "pathogenic" or "likely pathogenic" were grouped together, as were those annotated as "benign" or "likely benign," yielding a binary classification framework. Variants of uncertain significance (VUS), conflicting interpretations or lacking clinical annotation were excluded from this analysis, as their classification is inherently ambiguous.

Next, we evaluated the ability of cPRIME scores to dissect clinically pathogenic and benign variants using receiver operating characteristic (ROC) analysis. This analysis demonstrates that cPRIME scores show strong concordance with existing clinical annotations (AUC 0.98 and 0.99 for K562 and KBM7, respectively). Together, this provides support for the predictive utility of our approach (Fig. 4a). We note that our variant library is enriched for predicted splice-altering variants (based on Pangolin), and that clinical classifications are often informed by evidence of splicing disruption. Thus, some degree of concordance is expected. Importantly, this does not diminish the value of the assay, but rather highlights our ability to quantitatively resolve RNA-mediated pathogenic mechanisms. We further emphasize that cPRIME is specifically designed to measure effects on RNA abundance and processing. Thus, variants that affect protein function without altering RNA level mechanisms would not be expected to show strong depletion signatures, and their classification may require complementary functional assays. Thus, the predictive performance observed here is especially applicable to variants acting through RNA-level mechanisms.

We note that nonsense variants were excluded from this analysis, as their pathogenicity is typically well established due to their predictable impact on protein truncation and NMD of their RNA transcripts. Inclusion of these variants would therefore artificially inflate performance without providing meaningful insight into the discriminatory power of cPRIME for other types of variants.

These results have been added to the Results section and are supported by a new figure (Fig. 4), highlighting the translational relevance of our method for variant interpretation.

4. The authors state in Figure 3 that validation experiments were performed using cells edited with each mutation. However, simply relying on prime editing in a bulk population context is insufficient, as the editing efficiency of each pegRNA can vary substantially. Therefore, this approach may not provide adequate validation. More rigorously, it would be necessary to generate clonal cell lines harboring each individual variant and directly assess changes in RNA abundance—using methods such as qPCR or RNA-seq—rather than inferring effects from the cDNA/gDNA ratio used in the screening workflow.

We agree with the reviewer that clonal cell lines carrying individual variants, followed by orthogonal RNA-level measurements such as RT-qPCR or RNA-seq, would provide a rigorous approach for mechanistic validation of individual variant effects. However, for the variants tested here, the low absolute prime-editing efficiencies make the generation of clonal lines carrying each individual edit technically challenging and inefficient. Screening large numbers of clones for each variant would be required, particularly because the intended edits are often introduced at low frequencies.

Importantly, variation in pegRNA editing efficiency is accounted for by the cPRIME score calculation, which is based on paired quantification of the same edited variant in gDNA and cDNA. The gDNA measurement captures the editing frequency achieved by each pegRNA, whereas the cDNA measurement captures the representation of the same edited sequence at the RNA level. The resulting

cDNA/gDNA ratio therefore normalizes for differences in absolute editing efficiency between pegRNAs, provided that editing frequencies are above the defined detection threshold.

Minor points

1. The term "SNP" refers specifically to variants with a population frequency greater than 1%. Since the current study deals with individual nucleotide variants regardless of their population frequency, it would be more accurate to uniformly use the term "SNV" (single nucleotide variant) rather than "SNP" throughout the manuscript.

We agree with the reviewer that "SNV" is the more accurate term in this context, since the variants analyzed in this study are individual single-nucleotide variants and are not defined by population frequency. We have therefore revised the manuscript and now use "SNV" replacing previous instances of "SNP".

2. In Fig. 1h–j, the authors explore determinants of prime editing efficiency and comment on variables such as poly-T content and spacer sequence identity. However, there is already substantial literature systematically addressing the sequence features that impact prime editing efficiency—for example, GC content of the PBS or computationally predicted efficiency scores for the 20nt spacer (see, e.g., Yu et al., Cell, 2023, PMID: 37119812; Mathis et al., Nat Biotechnol, 2024, PMID: 38907037). Several algorithms have been developed to predict editing efficiency based on these parameters. To enhance the rigor and generalizability of these findings, I would recommend that the authors quantitatively analyze the correlation between their observed editing frequencies and the scores predicted by these established algorithms. Such analysis would provide more meaningful context for the observed variability in editing efficiency and more robustly inform future pegRNA library design strategies.

We thank the reviewer for this suggestion to relate our observed editing efficiencies to existing predictive tools.

In response, we applied PRIDICT2.0 to the same input sequences that were used for PrimeDesign. In total, 91 out of 98 variants got a successful prediction. We extracted predicted pegRNAs by PRIDICT2.0 that matched our predefined pegRNAs from the exon 27 screen. Specifically, we retained all pegRNAs with at most 1 mismatching base in the spacer sequence or extension sequence. After filtering we kept 88 pegRNAs for analysis. For these, we compared predicted editing scores to observed mean editing efficiencies across replicates derived from our exon 27 screen. As recommended by the authors of PRIDICT2.0 we compared our editing efficiencies to PRIDICT2_0_editing_Score_deep_K562 since our cell lines are mismatch repair (MMR) proficient.

We observed a spearman correlation of 0.73 and 0.67 for KBM7 and K562 cell lines, respectively (Reviewer Figure 1). This indicates that the predicted scores capture a substantial fraction of the variability in editing outcomes. This supports the relevance of previously described sequence features and predictive frameworks. At the same time, the correlation is not perfect, with considerable dispersion in observed efficiencies at similar predicted scores. This likely reflects additional factors influencing editing efficiency in our experimental setting, including constraints for example imposed by context-specific effects which are not yet fully captured by current models.

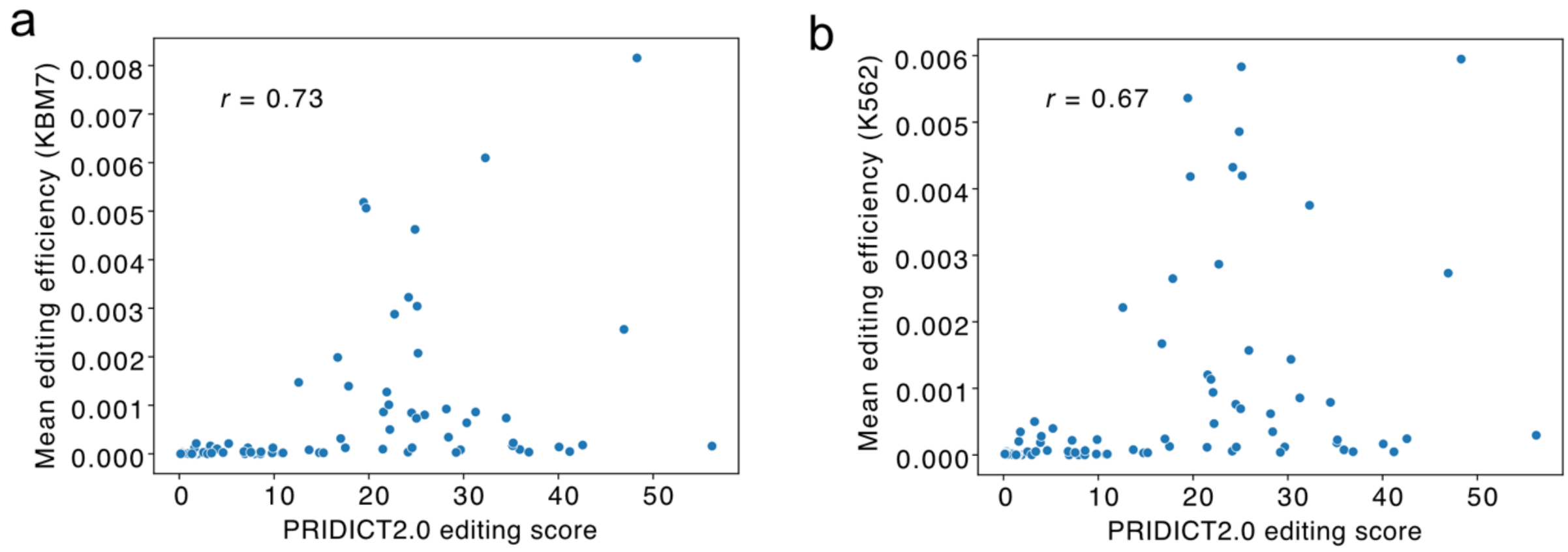

Reviewer Fig. 1 - PRIDICT2.0 prediction of pegRNA editing efficiency. a, Scatter plots comparing PRIDICT2.0-predicted editing scores with experimentally observed mean editing efficiencies for matched pegRNAs from the NF1 exon 27 screen in KBM7 and **b,** K562 cells. Observed editing efficiencies were calculated from dDNA sequencing counts as edited gDNA reads divided by unedited gDNA reads and averaged across replicate screens. Pearson correlation was used to determine the r value.

These results demonstrate that predictive algorithms like PRIDICT2.0 provide valuable guidance for pegRNA design, but empirical measurements remain important for fully resolving editing efficiency across diverse variant contexts.

Importantly, our analysis in Fig. 1i and 1j is not intended to provide a comprehensive predictive model, but rather to highlight specific sequence features that contribute to variability within a controlled pegRNA design framework. We agree that integration with predictive algorithms is an important future direction.

3. The authors state in Figures 3e and 3f that a reduction in RNA abundance was observed only when the intended edit was combined with the synonymous marker in the R1207R variant. Has the resulting sequence in these cases been reported as a motif known to induce changes in RNA splicing?

We thank the reviewer for this insightful question. In response, we examined the sequence context of the R1207R variant in more detail.

The wild-type sequence surrounding the edited region is: TGAGAGATTGGTGGAA whereas the sequence containing both the intended edit and the synonymous marker is: TGAGAG**G**TT**A**GTGGAA

Notably, the combined edit generates a sequence that closely resembles the canonical 5′ splice donor consensus motif. Specifically, the edited sequence contains the motif AGGTTAGT, which differs by only one nucleotide from the canonical donor-like motif AGGTAAGT. This near-consensus splice donor motif is generated only when both the intended edit and the synonymous marker are present, because both nucleotide changes contribute to conserved positions within the motif. Neither edit alone is sufficient to generate this sequence.

Consistent with this interpretation, Pangolin predicts a high splice-gain score for the combined edit (~0.75), whereas the individual edits or alternative markers show only minimal predicted splice gain (~0–0.2). To highlight this finding, we have added the corresponding Pangolin splice-gain predictions to Supplementary Fig. 1e,f.

4. On page 5, the authors claim: "Such an approach would eliminate the need for separate libraries, as required by SGE, and hence allow the simultaneous investigation of both exonic regions, as demonstrated in this study, and non-coding expressed regions such as untranslated regions (UTRs) or long non-coding RNAs." However, cPRIME relies on simultaneous sequencing

of target genomic DNA and cDNA, typically using Illumina platforms, which can generally capture sequences up to 300bp in length. If the targeted genomic region or the cDNA itself is substantially longer than this limit, the approach may still be constrained to analyzing short amplicons, and additional library preparations would be necessary for comprehensive coverage of longer regions. Supporting this concern, previous literature (e.g., PMID: 39533107) has shown that even for prime editing-based screens, separate libraries are often generated for individual exons, particularly when targeting large or multi-exon genes. Therefore, the claim that cPRIME universally eliminates the need for separate libraries is not fully convincing in practice, especially when analyzing long transcripts or large genomic regions. Given the method's reliance on high sequencing accuracy and short-read platforms, it is unclear that separate libraries can be universally avoided as the authors suggest.

We agree with the reviewer that this statement was too broad and could be interpreted as implying that cPRIME universally eliminates the need for region-specific amplicon-seq. We have revised the manuscript to clarify the current scope and limitations of the approach.

The main advantage of cPRIME is that variants targeting multiple genomic loci can be introduced in parallel using a single pooled pegRNA library, as is now demonstrated by the multi-exon screens. However, we agree that the sequencing-based readout remains constrained by amplicon design, sequencing read length, sequencing depth, and the need to quantify the same edited sequence in both gDNA and cDNA. Therefore, depending on the size and structure of the targeted region, separate PCR amplifications or sequencing preparations may still be required for comprehensive readout. We have revised the Discussion to emphasize that cPRIME enables pooled variant installation across multiple loci, but that the current implementation remains constrained by targeted gDNA/cDNA amplicon sequencing.

5. In the Methods section: "KBM7_PE_MLH1 cells were transduced with lentivirally packaged exon 27 library at multiplicity of infection (MOI) =0.7 and 100.000x cell coverage as triplicates..." In this context, do "100.000x" and "10.000x" refer to "100,000×" and "10,000×" respectively? The notation should be clarified for consistency with standard scientific convention.

We have revised the manuscript for consistency with standard scientific convention and now use commas as thousands separators throughout. For example, “100.000x” and “10.000x” have been corrected to “100,000×” and “10,000×”, respectively, throughout the Methods and the rest of the manuscript.

Reviewer #3 (Remarks to the Author):

Westphal et al. present a study using a small prime editing library to introduce variants in exon 27 of NF1. By comparing variant read counts from gDNA and cDNA, the authors measured both prime editing efficiency and the transcript-level consequences of editing. They validated that introducing nonsense or splice-disrupting variants reduces transcript abundance. The manuscript is clearly written, and the figures and data presentation are straightforward to follow. The attempt to incorporate transcript-level readouts in prime editing screening is interesting, and the discussion on the potential confounding effects of synonymous marker edits is a valuable contribution to the field. Nevertheless, while these aspects are useful, the overall cPRIME platform is constrained by low efficiency, small scale, and unclear advantages over existing methods.

Major comments:

1. The current work is restricted to ~100 pegRNAs targeting a single exon, with extremely low editing efficiencies. The methods are clearly described and appear reproducible, but in terms of target scope, time, and cost of screening, the study does not offer clear advantages over existing prime editing platforms (PMID: 38134886, 38472508, 39533107, 40580951). It would be helpful if the manuscript explicitly discussed potential distinctions and advantages compared to these existing platforms.

We would like to clarify that the main distinction of cPRIME is not enhanced prime-editing efficiency, but the coupling of pooled prime editing with matched gDNA and cDNA quantification to measure variant effects at the RNA level, including effects on RNA abundance, splicing, and nonsense-mediated decay. To our knowledge, the prime-editing studies cited by the reviewer (PMID: 38134886, 38472508, 39533107, 40580951) did not use a matched cDNA/gDNA readout to assess RNA-level variant effects. Instead, these studies primarily relied on DNA-level editing measurements or phenotypic readouts such as cell fitness, which often reflect altered protein function. By contrast, cPRIME directly measures the representation of endogenously introduced variants at the RNA level relative to their genomic abundance. This provides a conceptual and practical distinction from existing pooled prime-editing platforms. We have revised the manuscript to make this distinction more explicit.

2. Screening ~50 variants in a single exon is insufficient to support claims of a broadly applicable platform. At minimum, results from an additional exon or gene, or a concrete strategy for scaling to larger variant sets, would be necessary.

We agree that the initial screen of variants in a single NF1 exon was not sufficient on its own to support broad claims about the applicability of cPRIME. To address this concern experimentally, we performed an additional cPRIME screen targeting five further NF1 exons together with the original exon 27, using a single pooled library of 341 pegRNAs. This expanded screen included 178 intended edits distributed across six NF1 exons and demonstrates that cPRIME can be applied beyond a single exon to assess variants across multiple genomic regions in a pooled format.

We have incorporated these new data into the revised manuscript and now present the expanded NF1 multi-exon screen as evidence that cPRIME can be scaled to defined sets of genomic regions. At the same time, we have revised the text to avoid overstatement and now emphasize that the current implementation of cPRIME is best suited for targeted pooled interrogation of selected loci. Broader scaling will depend on factors such as editing efficiency, pegRNA design, sequencing depth, and the feasibility of matched gDNA/cDNA amplicon-based readouts.

3. The use of cDNA readouts to analyze RNA metabolism (e.g., NMD, splicing) is conceptually novel in prime editing screening. However, the contribution remains limited, as prior MPRA-based approaches (PMID: 35051175, 37192163, 40715118) have already enabled high-throughput splicing variant analysis. To my knowledge, chromatin status has little impact on splicing outcomes, making it unclear whether cPRIME provides meaningful advantages over MPRA. In fact, cPRIME requires the generation of PE-expressing, MMR-deficient cell lines, which may make the system more complex rather than more practical. A direct performance comparison with MPRA would strengthen the manuscript.

We agree that MPRA-based approaches have enabled powerful high-throughput analyses of splicing variants and that cPRIME should not be presented as replacing these methods. We have therefore revised the manuscript to more clearly position cPRIME as a complementary approach. The key distinction is that cPRIME measures the consequences of variants introduced at their endogenous genomic loci, whereas MPRA relies on reporter constructs outside the native genomic and transcript context. This allows cPRIME to preserve the endogenous exon–intron architecture, local sequence context, transcriptional environment, and native mRNA processing and turnover pathways.

While chromatin status may not be the dominant determinant for all splicing events, splicing is often coupled to transcription and can be influenced by endogenous gene architecture, transcript context, and RNA processing kinetics. In addition, cPRIME directly measures the relative abundance of edited variants in cDNA compared with gDNA, thereby capturing RNA-level consequences such as splice-associated transcript depletion and nonsense-mediated decay in the context of the native transcript. These features make cPRIME particularly useful for prioritizing variants whose effects depend on endogenous RNA processing or transcript stability.

We also agree that the requirement for prime-editor-expressing cells, and in our current implementation MLH1 loss to enhance editing efficiency, adds experimental complexity compared with reporter-based assays. We now acknowledge this limitation more explicitly in the Discussion. However, once established, these cell systems can be used for pooled interrogation of endogenous variants without constructing locus-specific reporter libraries. Therefore, rather than serving as a direct substitute for MPRA, cPRIME provides a complementary strategy for validating and prioritizing RNA-altering variants in their native genomic context. A systematic head-to-head comparison with MPRA would be valuable in the future but is beyond the scope of the present study.

cPRIME enables endogenous mapping of genetic variant effects at RNA level

Ghanem El Kassem[1*], Constanze Westphal[1*], Marvin Doebel[1*], Jasmine Hillmer[1*], Tanja Seifert[1], Michael Boettcher[1#]

[1] Institute of Molecular Medicine, Section for Molecular Medicine of Signal Transduction, Faculty of Medicine, Martin Luther University Halle-Wittenberg, 06120 Halle, Germany

[#]Correspondence to: michael.boettcher@medizin.uni-halle.de

*equal contribution

We would like to thank the reviewers for their careful evaluation of our manuscript and for their constructive comments, which helped us substantially improve the scope, clarity, and interpretation of the study. Below, we respond point by point to the reviewers' comments.

REVIEWER COMMENTS

Reviewer #1 (Remarks to the Author):

The authors have done well to revise their manuscript. Most importantly, they've added a considerable amount of data in a new experiment, showing that their approach can be applied to multiple exons at the same time, which constitutes a unique advantage of cPRIME. Thanks for taking the reviewer feedback onboard and improving the manuscript.

The addition of new data is much appreciated, but the major limitation of the manuscript is still the relatively low throughput of the experiments compared to other methods. The maximum library size remains under 200 variants / 400 pegRNAs. Therefore, while the technical quality of the data looks alright, it's still not established to what extent cPRIME may be scaled up further. Despite this major limitation, I find the method's strengths and weaknesses to be well characterised within the context of the current literature. Therefore, with some additional revisions I would recommend this for publication.

1. Regarding "Within the cPRIME workflow, such transcripts may be underrepresented during cDNA amplification and amplicon purification, for example due to loss of primer binding sites or the generation of shorter splice isoforms.":

Please specify here exactly how the cPRIME score is calculated. Specifically, if a variant is observed in cDNA on an alternative splice form (arising due to altered splicing), do those reads contribute to the variant's cPRIME score, or are only reads used if they map to the canonically spliced transcript? I ask because in many use cases it will be important to know whether low cPRIME scores reliably mean loss-of-function due to low transcript levels, or whether alternative "rescue" transcripts may arise that will not be accounted for in the score.

We thank the reviewer for this point, and in response, we have clarified both how the score is calculated and what a reduced score does and does not imply. The cPRIME score is the ratio of cDNA to gDNA RPM for each intended edit, where reads are counted only if they contain an intact search sequence consisting of the intended edit, its synonymous marker, and 10 bp of flanking reference sequence. Because the cDNA amplicon is generated with primers in the neighboring exons and is size-selected by gel purification before library preparation, a variant-bearing transcript contributes to the cDNA count only when it retains this search sequence, both flanking primer binding sites, and an approximately canonical amplicon size. Transcripts in which the variant activates a cryptic splice site typically fail one or more of these criteria, because the cryptic event disrupts the search sequence and/or changes the amplicon size, and are therefore not counted, so the variant shows a reduced score. An alternative isoform that happened to preserve the search sequence, the primer binding sites, and the canonical amplicon size would still be detected and would not lower the score.

A reduced cPRIME score therefore reflects depletion of the specifically detected, canonically spliced, variant-bearing isoform rather than necessarily a reduction in total transcript levels. For nonsense variants this corresponds to genuine transcript loss through nonsense-mediated decay, whereas for splice-altering variants, the variant-bearing RNA may be rerouted into an alternative isoform that escapes amplification, in which case the canonical isoform is lost from the assay even though variant-bearing RNA persists in the cell.

cPRIME thus reliably identifies variants that reduce the abundance of the canonical transcript, but does not by itself establish whether an alternative isoform is produced or whether it is functional; as we note in the Discussion, resolving this requires isoform-resolved assays such as isoform-specific RT-PCR or long-read transcript sequencing. We have added text to the Results to state explicitly how the score is calculated and what a reduced score does and does not imply.

2. Sample sizes are required throughout Figure 4 to place the performance metrics in context. For instance, it appears as if the ROC-AUC analysis was performed using very few gold-standard variants, based on the following panels. This is essential information, so please add n = x for each group to the figures themselves.

In response to this comment, we have added the sample size of each group to the corresponding panels of Figure 4. For the ROC analyses, we used variants that passed the RPM cutoff of 5 and carried a (likely) benign or (likely) pathogenic ClinVar annotation. Also, we computed cPRIME scores by averaging across replicates and then across synonymous marker variants for each intended edit, while excluding measurements whose marker edits substantially altered the Pangolin score of the intended variant alone. The group sizes differ between panels because the ROC analysis in Figure 4a includes only ClinVar pathogenic/likely pathogenic and benign/likely benign variants, whereas the distributions in Figure 4 b,c additionally include variants of uncertain significance and variants with conflicting classification. Accordingly, the ROC analysis is based on 3 pathogenic and 17 benign variants in both cell lines, and the distributions in Figure 4 b,c have n = 38 (KBM7) and n = 36 (K562) non-STOP edits.

During this revision, we also updated the ROC analysis to exclude nonsense variants, which had been retained in the original calculation despite the exclusion rationale described in the Methods. Because the pathogenicity of nonsense variants is primarily driven by protein truncation, their inclusion could overestimate classification performance. After careful revision, the cPRIME score still separated the available pathogenic and benign ClinVar variants (AUC = 1.0). Importantly, we emphasize in the manuscript that this analysis is based on a small set of gold-standard ClinVar variants and therefore we present the ROC analysis as supporting evidence for the cPRIME-score

distributions shown in Figure 4b,c rather than as a definitive performance benchmark. Finally, we modified the figure legend to better indicate which variant classes were used in each analysis.

3. Related to the point above, I was surprised to see the method work so well for ClinVar Pathogenic variants because many pathogenic variants will not impact splicing (for instance, most missense variants). Reading the methods, it became clear that variants were pre-selected for testing due to potentially having an impact on RNA levels. Therefore, please make it explicit in the Results which ClinVar variants were included in testing, and what the criteria was applied to prioritise them.

We agree that the selection strategy for ClinVar pathogenic variants should be stated more explicitly and prominently. The variants included in this analysis were not intended to represent an unbiased sample of all ClinVar variants. Rather, we first prioritized genomic positions at which at least one variant was predicted by Pangolin to result in splice gain activity, with a splice loss being smaller than the splice gain and where the splice effect was located at least 10 bp apart from the next canonical splice site. We reasoned that these positions have a good potential to show a strong effect in cPRIME. At these selected positions, we included all SNVs that either showed a predicted splice gain by Pangolin or were listed in ClinVar. This strategy ensured that the analyzed variants were located in regions with predicted splice potential, thus, being consistent with the biological processes measured by cPRIME. We have now clarified the selection criteria in the Results and Material & Methods sections.

4. The authors attempt to classify variants into one of three functional categories using the GMM. What was the rationale for having 3 categories instead of 2? It seems most critical to establish the score threshold below which variants are highly likely to be pathogenic, in line with an ACMG-style calibration (Brnich et al 2019). Currently, the "responder" category seems to include only variants with a cPRIME score < -2 (log10 scale), corresponding to a 99% depletion in RNA. Is this correct? This seems much lower than what may be necessary for identifying pathogenic, splice-disruptive variants. Meanwhile the intermediate category is quite broad and includes far more variants (20/15 vs 7). Not sure whether this was the case here, but as nonsense variants impact function via premature protein truncation, these should be excluded from any analysis that attempts to calibrate the scores to clinical pathogenicity.

We thank the reviewer for this valuable comment on the interpretation of the GMM analysis. We agree that nonsense variants should not be used to calibrate ACMG-style pathogenicity thresholds for splice-disruptive variants. However, the purpose of this analysis was not to define clinically calibrated pathogenicity thresholds. Rather, the GMM was used as an unsupervised approach to identify distinct cPRIME response classes within the experimental data. Thus, the resulting categories represent empirically derived groups. The number of components was selected based on the BIC criterion, which supported a three component model (Supp. Fig. 6).
Because the GMM analysis aims to capture experimentally observed groups rather than establish pathogenicity thresholds, nonsense variants were retained in this unsupervised classification. We have clarified this rationale in the revised text and now explicitly state that these categories should be interpreted as cPRIME response clusters rather than clinically calibrated pathogenicity cutoffs.

As part of the revised analysis workflow, we regenerated the GMM analysis using the corrected variant-level dataset, which did not affect the resulting cPRIME response classes or their interpretation. Additionally, to indicate the contribution of nonsense variants in the GMM, we revised Figure 4 panel h to show the proportion of nonsense variants within each class.

5. I found the contrast between black and gray difficult to appreciate in Fig3f. Further, it would be good to use a scheme that also discriminates which variants were hits (as in d, e), because ultimately the reader is keen to know how this impacts hits.

We have replaced the black/gray theme with red/black to improve contrast, and the legend now reads: "Relationship of Pangolin scores between multi-exon screen NF1 variants with and without marker edits. Marker edits whose predicted Pangolin score is altered by the marker by $|\Delta\text{Pangolin}| < 0.2$ are shown in red (non-confounding markers)." We have also added the threshold $|\Delta\text{Pangolin}| < 0.2$ directly to the figure to indicate what the red data points represent.

Regarding the suggestion to also indicate hits in this panel, the purpose of Figure 3f is to visualise how strongly each synonymous marker shifts the Pangolin-predicted splice effect of its intended edit, and thereby to define the non-confounding markers (red) that we included in the downstream ROC and ClinVar analyses, as described in the Results. To show how marker confounding affects the variants of interest, the per-variant impact is shown in Supplementary Figure 5, which displays Pangolin and cPRIME scores for each intended edit separately for its confounding and non-confounding markers for variants passing the RPM > 5 cutoff. Excluding the confounded markers before classification (Fig. 4) prevents marker-induced splice effects from being attributed to the intended edit.

6. I would certainly change the "responder" vs. "non-responder" terminology to something more consistent with established ways of describing variant effects. For instance, "RNA-depleted", "RNA-intermediate", "RNA-neutral", maybe? (To me, 'Responder' sounds like it relates to a drug response.)

We agree that "responder/non-responder" carries an unintended drug-response connotation. We have adopted RNA-based terminology consistent with the reviewer's suggestion. We have updated this terminology throughout the Results, Methods and Figure 4 (panels d–h) accordingly.

Reviewer #2 (Remarks to the Author):

Overall, the revised manuscript has been substantially improved, and I appreciate the authors' efforts to address the previous comments. In particular, the additional multi-exon analysis, the clearer discussion of marker-dependent effects, and the introduction of a quantitative classification framework have strengthened the study. I do not think additional experiments are necessary at this stage, but I have a few suggestions that may further improve the clarity and completeness of the manuscript.

1. Regarding the statements, "By eliminating the need for locus-specific DNA donor template pools required for SGE, cPRIME enables variants across multiple genomic loci to be installed from a single pegRNA library" and "Thus, cPRIME enables pooled installation and screening of variants across multiple genomic loci using a single pegRNA library, while the sequencing readout remains target-specific and must be adapted to the genomic and transcript architecture of the region under investigation."
These revisions are helpful and provide a more balanced description of the current scope of the method. At the same time, it would be useful to discuss more explicitly the trade-off between breadth and effective per-target coverage in the multi-exon format. As shown in Fig. 3c, the fraction of variants detected above threshold remains relatively modest and uneven

across exons, ranging from 7.3% to 38% in KBM7 cells and from 7.3% to 34% in K562 cells. This suggests that, as library complexity increases, effective coverage for individual target regions may become limiting, particularly given the low and variable prime-editing efficiencies observed here. In such settings, region-specific or exon-focused libraries may be preferable in some cases to help preserve coverage and sensitivity. A somewhat fuller discussion of this trade-off would strengthen the manuscript.

We thank the reviewer for this helpful point and have revised the Discussion to address the breadth-coverage trade-off more explicitly. We clarify that the modest and uneven per-exon detection is primarily driven by the low and variable target-site-specific editing efficiency rather than by overall coverage. Since a variant's expected representation scales with its editing efficiency and library size, the required coverage is dictated by the least efficient pegRNAs, which set the depth needed to clear threshold while the same coverage "over-covers" more efficient pegRNAs by orders of magnitude. We now also note that the underlying cause for this issue, namely low and variable prime-editing efficiency, is being progressively addressed by improved pegRNA design and next-generation prime editors, which are expected to lower the coverage needed per pegRNA.

2. The Pangolin-based analyses are a useful part of the manuscript and help interpret many of the cPRIME score hits. Nevertheless, because the study relies so heavily on a single splice predictor, it would further strengthen the manuscript if the authors could show that similar conclusions are supported by at least one independent predictor, such as SpliceAI (PMID: 30661751). This would help demonstrate that the main interpretations are robust to predictor choice rather than being specific to Pangolin.

Following the reviewer's suggestion, we have added an independent splice-prediction analysis using SpliceAI (new Supplementary Fig. 4). Across all multi-exon pegRNAs, SpliceAI and Pangolin scores are strongly correlated ($r = 0.93$), indicating that the splice-effect predictions underlying our interpretation are not specific to Pangolin. Variants identified as significant cPRIME hits (cPRIME score < 1 and nominal $p < 0.05$; red in Fig. 3d,e) are highlighted in red in this figure and show that the agreement between predicted splice effects and experimentally measured cPRIME scores is reproduced when Pangolin is replaced by SpliceAI. We have added a description of this analysis to the Results and a corresponding Methods section detailing the SpliceAI version, annotation and score definition used.

3. Regarding the statement, "The library showed a narrow distribution, with 96.8% of pegRNAs falling within one order of magnitude (Supp. Fig. 2d)."
The intended meaning of this sentence remains somewhat unclear. It would be helpful if the authors could clarify in the Results text what the normalized read counts refer to, what sample or library was sequenced to obtain them, and how the "within one order of magnitude" criterion was defined. Making the corresponding figure legend more explicit would also improve readability.

The text, Figure 1e and Supplementary Figure 2d refer to the representation of pegRNAs in the plasmid libraries used throughout the screens. To measure this, the pegRNA cassette was amplified from the plasmid pool using indexed primers compatible with Illumina sequencing, and the amplicon was sequenced. For each pegRNA, the read count was normalized in two ways for the plot. First, the x-axis shows the normalized read count, defined as each pegRNA's count divided by the mean count across all pegRNAs, and the y-axis shows the cumulative fraction of pegRNAs, obtained by dividing each count by the summed counts of all pegRNAs and accumulating these fractions from lowest to highest. The statement "within one order of magnitude" refers to the x-axis, where 96.8%

of pegRNAs have mean-normalized counts within a single 10-fold window (0.24 to 2.4 relative to the mean. The window is represented by dashed lines in Supp. Fig. 2d), indicating a narrow and even library representation. We have revised the Results and the legends of Figure 1e and Supplementary Figure 2d to state explicitly what the normalized counts represent, which sample was sequenced, and how the criterion was defined.

4. The terminology should be revised consistently throughout the manuscript. “SNP” still appears in Fig. 1c and the Methods, despite the previous request to replace this term with “SNV.”

We thank the reviewer for catching this. We have now performed a complete pass over the manuscript, including all figure panels, the Methods text and formulae, and the supplementary tables and figures, and have replaced every remaining instance of “SNP” with “SNV”.

5. Regarding the statement, “Interestingly, several variants, most notably, A1071E_G>A (edit: c.3212C>A, marker: c.3210G>A) and A1071V_G>A (edit: c.3212C>T, marker: c.3210G>A) showed significantly increased cPRIME scores in both cell lines, suggesting increased relative abundance of the edited transcript (Fig. 3c). Additional experiments would be required to illuminate the underlying mechanism.”
These observations are intriguing. While additional mechanistic experiments are likely beyond the scope of the present study, it would strengthen the manuscript if the authors could provide a somewhat fuller discussion of plausible explanations, such as altered RNA processing, increased transcript stability, or possible marker-related effects, and indicate whether any in silico analyses support these hypothesis.

We agree with the reviewer that these observations are intriguing and, in response, we have expanded our results section to include a new in silico analysis (new Supplementary Fig. 3). Pangolin did not predict a splice-site gain for either edit-marker combination (Pangolin gain score ≤ 0.02), making altered splicing an unlikely explanation. We further evaluated the variant sequences with the m6A site predictor deepSRAMP (PMID: 39571573), which predicted an increased probability of an m6A site for both variants relative to wild type (wild type 9%, A1071E_G>A 24%, A1071V_G>A 38%). However, we interpret this cautiously since m6A can either stabilize or destabilize transcripts depending on the RNA-binding proteins involved, and although it more frequently promotes decay, stabilizing effects e.g. through IGF2BP-family members are well documented. The predicted gain of an m6A site is therefore a plausible sequence-level basis for the altered transcript abundance but does not by itself establish the direction or mechanism of the effect, which would require experimental validation such as m6A mapping and RNA-stability assays. We have added these results as a new supplementary figure 3 and revised the Results accordingly.

6. The Gaussian mixture model-based classification is a useful addition. For reproducibility, the Methods should specify the covariance structure used for the GMM, for example whether the components were modeled with shared or component-specific covariance.

We thank the reviewer for this comment on the GMM analyses. Models were fitted using the GaussianMixture implementation from scikit-learn. The default covariance structure (covariance_type=”full”) was used, allowing each component to have its own variance. The details were also added in the Methods section of the manuscript.

Reviewer #3 (Remarks to the Author):

In the revised manuscript, the authors have thoroughly addressed my concerns through additional wet-lab experiments and computational analyses. In particular, the multi-exon screen and the comparison with ClinVar pathogenicity annotations substantially strengthen the claim of general applicability, and the repositioning of cPRIME relative to existing platforms is now much clearer. This work would be highly beneficial for a wide range of readers in the functional genomics and variant interpretation field.
